# Spatiotemporal patterns and county-level coordination between sports-related intangible cultural heritage and tourism development in the Wuling Mountain Area, China

**DOI:** 10.1371/journal.pone.0362452

**Published:** 2026-11-11

**Authors:** Jiaxin Zhang, Siqing Zhang, Junrong Lei

**Affiliations:** 1 College of Physical Education, Hunan Normal University, Changsha, Hunan, China; 2 School of Physical Education, University of South China, Hengyang, Hunan, China; 3 Department of Physical Education, Zhejiang Chinese Medical University, Hangzhou, Zhejiang, China; Central University of Finance and Economics, CHINA

## Abstract

Sports-related intangible cultural heritage (SRICH) can support culturally grounded tourism, but its county-level alignment with tourism development remains unclear in ethnically diverse mountain regions. This study examined 71 county-level units in China’s Wuling Mountain Area in 2010, 2015, 2019, and 2024. Geographical concentration, kernel density estimation, and standard deviational ellipses traced the administrative distribution of officially recognized SRICH records. Entropy weighting, coupling coordination, relative-balance diagnosis, and Moran’s I assessed their county-level relationship with tourism development. SRICH coverage expanded and geographical concentration declined, while county-represented density peaks increased within a broadly stable regional configuration. Tourism development rose rapidly before 2019 and more slowly thereafter, retaining a marked core–periphery structure. Mean D increased from 0.192 to 0.403; 62.2% of this change was associated with counties entering the official inventory after 2010. Within the baseline-covered cohort, the joint levels of the two subsystems increased, whereas relative balance changed little, and SRICH-lagging counties remained predominant. All three indices showed significant positive global spatial autocorrelation, while FDR-adjusted local clusters were sparse and none remained significant in 2024. The results inform differentiated cross-provincial safeguarding and tourism planning, with greater attention to counties where the two subsystems follow different trajectories. The coupling coordination degree D is interpreted as a comparative measure of the recorded joint levels of SRICH and tourism development.

## 1. Introduction

The safeguarding of intangible cultural heritage has gradually entered the broader agendas of sustainable development and local governance [1]. The UNESCO Convention for the Safeguarding of the Intangible Cultural Heritage defines ICH as living heritage that communities and groups transmit across generations and continually recreate in response to their social environment. This definition directs attention to heritage bearers, cultural spaces, and the processes through which cultural practices remain viable [2]. The 2030 Agenda for Sustainable Development further connects cultural safeguarding with sustainable tourism, employment creation, and the monitoring of development impacts [3]. Correspondingly, the Operational Directives for the Implementation of the Convention call on states to assess the effects of tourism on heritage viability and to secure the effective participation of heritage communities in tourism planning, presentation, and benefit-sharing [4]. Sustainable tourism use must therefore preserve the conditions under which heritage is practiced, transmitted, and interpreted by its communities.

China has developed a multilayered, state-led system for ICH safeguarding through representative inventories, designated inheritors, and administrative implementation at national and subnational levels [5,6]. The Intangible Cultural Heritage Law of the People’s Republic of China, enacted in 2011, provides the principal legislative basis for this system and formally incorporates traditional sports and recreational practices into the protected heritage domain. Traditional sports-related practices consequently occupy an identifiable position within China’s safeguarding framework, with recognition, documentation, and protection administered by different levels of government, including county-level cultural authorities.

Sports-Related Intangible Cultural Heritage (SRICH) warrants separate examination because its cultural knowledge is embodied in movement techniques, bodily memory, competition rules, ritual procedures, and collective performance. Its continuity depends heavily on demonstration, imitation, repeated practice, and community participation. These characteristics enable SRICH to connect local history and ethnic memory with visitors’ bodily and sensory experiences, providing place-specific content for festivals, sporting events, educational travel, and rural recreation [7,8]. Ramshaw and Gammon describe sports heritage as a dynamic resource shaped through place, tradition, memory, and participation, whose tourism value relies on continued practice and reinterpretation [9]. Its performative visibility also makes it particularly susceptible to tourism-led reconfiguration. Staged presentations may shorten ritual sequences or separate them from community calendars, while market adaptation often favors recognizable movements over locally situated meanings [10,11]. The involvement of external performers may further weaken the interpretive authority of heritage bearers and turn living practices into standardized tourism products [12]. In response to these risks, research in China has emphasized that tourism use should be assessed in relation to the cultural setting of each practice and should involve practitioners and other local stakeholders in planning and value creation [13]. Evidence also shows that market-led heritage production can distance practitioners from the cultural meanings of their activities and weaken their sense of authenticity and ownership [14].

The Wuling Mountain Area provides an analytically revealing setting in which to examine these relationships. Extending across Hunan, Hubei, Chongqing, and Guizhou, the region forms a mountainous cultural corridor shaped by long-term interaction among Tujia, Miao, Dong, and other ethnic communities. Cultural connections extend across provincial boundaries, whereas heritage recognition, fiscal expenditure, and tourism provision are administered largely through county-level units. Cultural continuity thus coexists with administrative fragmentation [15,16]. The region was incorporated into the Wuling Mountain Area Regional Development and Poverty Alleviation Plan (2011–2020), under which tourism was expected to support regional coordination and local development. Yet the spatial distribution of officially recognized SRICH does not correspond uniformly with tourism development. Some counties possess substantial heritage endowments but limited infrastructure and service capacity; others have mature tourism economies but comparatively few officially recognized sports heritage projects [17]. Heritage endowment and tourism capacity may therefore be geographically proximate while developing at different rates. Examining this relationship is necessary for identifying where safeguarding priorities and tourism investment remain spatially misaligned [18].

International research has developed several important perspectives on heritage tourism, particularly heritage production, authenticity, community participation, and commercialization. Kirshenblatt-Gimblett interprets the incorporation of intangible heritage into exhibition and tourism systems as a form of institutionalized cultural reproduction [19]. Cohen shows that commodification reshapes the definition and negotiation of authenticity, with outcomes that vary across social and cultural settings [20]. From the perspective of practitioners, Kim and colleagues identify community interests, transmission conditions, and cultural integrity as essential constraints on the development of sustainable ICH tourism [21]. Chinese scholarship has advanced along two main methodological lines. One employs kernel density estimation, standard deviational ellipses, and spatial autocorrelation to examine concentration centers, directional change, and tourism utilization potential [7,17,22–24]. The other constructs subsystem indices and applies coupling models to evaluate the relative alignment of heritage resources and tourism development [25–29].

Several aspects of this literature require further attention. Studies frequently combine different ICH categories into a single dataset, despite substantial differences in their spatial configurations and formation mechanisms [30]. This aggregation is particularly consequential for traditional sports and games because their continuity depends on embodied competence, collective enactment, and sustained relationships with particular social and environmental settings [31,32]. Project counts and registered locations provide useful evidence of formal heritage recognition, but they capture these cultural qualities only indirectly. Analytical scale presents a related problem. Provincial- and prefectural-level studies may conceal marked variation among counties, and their results remain sensitive to the scale and zoning through which spatial data are aggregated [33]. County-level analysis offers a more suitable resolution for the present study. China’s representative-list system operates across national, provincial, municipal, and county levels, while patterns of recognition and tourism development may vary considerably within the same prefecture [34]. The county scale also allows officially recognized heritage endowment to be compared with locally differentiated tourism capacity on a consistent spatial basis. Resource concentration and coupling coordination must also be distinguished conceptually. Concentration measures describe the spatial clustering or dependence of observed resources. Coupling coordination models assess the relative configuration of standardized subsystem indices and are sensitive to indicator construction, weighting, and model specification [35]. Neither type of measure establishes a causal relationship between heritage and tourism. Nor can it independently demonstrate cultural vitality, community welfare, environmental responsibility, or sustainable development, all of which require broader multidimensional evidence [36]. An integrated analysis should therefore connect project-level spatial patterns with county-level subsystem conditions while maintaining clear limits on what each indicator can support.

Accordingly, this study takes the 71 county-level administrative units of the Wuling Mountain Area as its empirical setting and integrates officially recognized SRICH records with county-level tourism data for 2010, 2015, 2019, and 2024. Project-level spatial methods, including the geographical concentration index, kernel density estimation, and standard deviational ellipses, are combined with county-level coupling coordination, relative-balance, and spatial autocorrelation analyses to trace the spatiotemporal evolution of SRICH and assess its alignment with tourism development. By identifying areas where heritage endowment and tourism capacity follow divergent trajectories, the study seeks to deepen understanding of their spatial relationship and to inform differentiated safeguarding and tourism development strategies for cross-provincial ethnic mountain regions.

## 2. Materials and methods

### 2.1 Study area

The Wuling Mountain Area (WMA) lies between 107°–111°E and 27°–31°N, extending across Hunan, Hubei, Chongqing, and Guizhou in the transitional zone between central and southwestern China. It covers approximately 171,800 km² and comprises 71 county-level administrative units under 11 prefecture-level jurisdictions (Fig 1 and Table 1) [37]. Predominantly mountainous, the region has an average elevation above 1,000 m, a subtropical monsoon climate, and extensive forest cover, conditions that support high ecological value and mountain-based ecotourism. More than 30 ethnic groups, including the Tujia, Miao, Dong, Gelao, Bai, and Yao, inhabit the area, with ethnic minorities accounting for over 40% of the population. Prolonged interethnic interaction and the relative isolation of mountain settlements have produced a distinctive cultural ecology [38].

**Table 1:** County-level administrative units included in the Wuling Mountain Area.

Provincial-level region	Prefecture-level jurisdiction	County-level administrative units (administrative division codes)	No.
Hunan Province	Xiangxi Tujia and Miao Autonomous Prefecture	Jishou City (433101); Luxi County (433122); Fenghuang County (433123); Huayuan County (433124); Baojing County (433125); Guzhang County (433126); Yongshun County (433127); Longshan County (433130)	8
Hunan Province	Zhangjiajie City	Yongding District (430802); Wulingyuan District (430811); Cili County (430821); Sangzhi County (430822)	4
Hunan Province	Huaihua City	Hecheng District (431202); Zhongfang County (431221); Yuanling County (431222); Chenxi County (431223); Xupu County (431224); Huitong County (431225); Mayang Miao Autonomous County (431226); Xinhuang Dong Autonomous County (431227); Zhijiang Dong Autonomous County (431228); Jingzhou Miao and Dong Autonomous County (431229); Tongdao Dong Autonomous County (431230); Hongjiang City (431281)	12
Hunan Province	Shaoyang City	Xinshao County (430522); Shaoyang County (430523); Longhui County (430524); Dongkou County (430525); Suining County (430527); Xinning County (430528); Chengbu Miao Autonomous County (430529); Wugang City (430581)	8
Hunan Province	Changde City	Shimen County (430726)	1
Hunan Province	Yiyang City	Anhua County (430923)	1
Hunan Province	Loudi City	Xinhua County (431322); Lengshuijiang City (431381); Lianyuan City (431382)	3
Hubei Province	Yichang City	Zigui County (420527); Changyang Tujia Autonomous County (420528); Wufeng Tujia Autonomous County (420529)	3
Hubei Province	Enshi Tujia and Miao Autonomous Prefecture	Enshi City (422801); Lichuan City (422802); Jianshi County (422822); Badong County (422823); Xuan’en County (422825); Xianfeng County (422826); Laifeng County (422827); Hefeng County (422828)	8
Guizhou Province	Zunyi City	Zheng’an County (520324); Daozhen Gelao and Miao Autonomous County (520325); Wuchuan Gelao and Miao Autonomous County (520326); Fenggang County (520327); Meitan County (520328); Yuqing County (520329)	6
Guizhou Province	Tongren City	Bijiang District (520602); Wanshan District (520603); Jiangkou County (520621); Yuping Dong Autonomous County (520622); Shiqian County (520623); Sinan County (520624); Yinjiang Tujia and Miao Autonomous County (520625); Dejiang County (520626); Yanhe Tujia Autonomous County (520627); Songtao Miao Autonomous County (520628)	10
Chongqing Municipality	Directly administered county-level units	Qianjiang District (500114); Wulong District (500156); Fengdu County (500230); Shizhu Tujia Autonomous County (500240); Xiushan Tujia and Miao Autonomous County (500241); Youyang Tujia and Miao Autonomous County (500242); Pengshui Miao and Tujia Autonomous County (500243)	7

**Fig 1:**
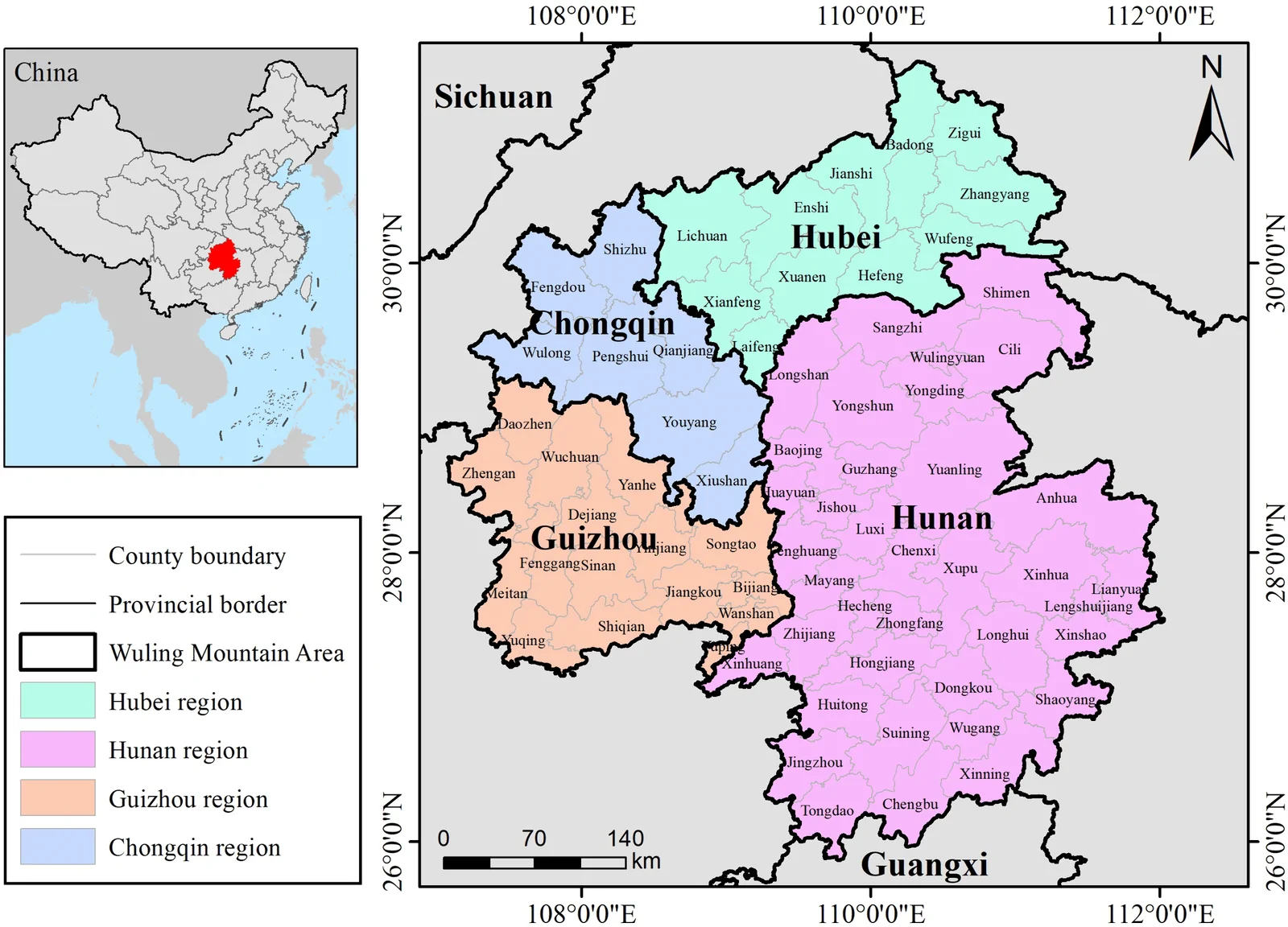
Location map of Wuling Mountain Area. Note: Administrative boundary data were obtained from the Standard Map Service System of the China Ministry of Natural Resources (http://bzdt.ch.mnr.gov.cn/), under the terms stated on the service website. The maps were created and edited by the authors. The administrative boundary data correspond to map review number GS(2024)0650. No Google Maps, Google Earth, Street View, satellite imagery, or other proprietary basemap was used.

Historically, the WMA formed part of the Wuling Ethnic Corridor linking the Central Plains with southwestern China. The tusi system, its later reform, successive population movements, and adaptations to mountain agriculture shaped the coexistence of diverse rituals, festivals, crafts, and embodied traditions [15,39]. Official inventories compiled up to December 2024 recorded about 126 national-level and 726 provincial-level representative ICH entries across the 71 counties, covering traditional music, dance, drama, crafts, folk customs, and traditional sports and games. Regionally distinctive practices such as the Baishou dance, Maogusi, and Miao drum dance remain embedded in village festivals, ritual life, and collective participation [40], making the region an important setting for examining the spatial distribution and institutional recognition of living heritage.

The WMA was designated in 2011 as one of China’s 14 contiguous poverty-stricken areas owing to its weak economic base, difficult terrain, and widespread county-level poverty [41,42]. Absolute poverty has since been eliminated under the national standard, but development remains uneven and rural revitalization continues to be a major concern [43]. Tourism has accordingly been promoted as a strategic industry, with destinations such as Wulingyuan, Fanjingshan, and Tianmen Mountain stimulating growth in several counties, yet marked differences in accessibility, infrastructure, and service capacity persist [44]. SRICH provides locally rooted and participatory content for festivals, village-based experiences, and educational travel, but counties differ considerably in their ability to safeguard, interpret, and support these practices through tourism. The combination of abundant sports heritage, strong tourism-policy orientation, ecological constraints, and pronounced county-level disparities makes the WMA a suitable case for examining the spatiotemporal distribution of SRICH and its relative alignment with tourism development.

### 2.2 Conceptual definition and analytical classification of sports-related intangible cultural heritage

Since the first national representative list was published in 2006, China has classified intangible cultural heritage (ICH) into ten official categories. In 2008, “acrobatics and competitive sports” was renamed “traditional sports, recreation and acrobatics,” a designation retained in subsequent national lists [40,45]. Sports-related intangible cultural heritage (SRICH), however, is not an independent category within this official system. Relevant practices may appear not only in traditional sports, recreation and acrobatics but also within dance, performance, festivals, and folk customs. An operational definition was therefore required to identify sports-related practices consistently without altering China’s official ICH classification.

This study defined SRICH as officially recognized intangible cultural heritage in which structured bodily practice is integral to the item’s form, transmission, or cultural meaning. The definition follows scholarship that treats traditional sports, games, and performances as embodied cultural practices involving physical skills, rules, collective participation, and culturally situated transmission [32,46]. Eligible practices were those containing identifiable sport-related elements, including transmissible physical skills, rule-governed games or competition, systematic training, or codified collective performance. Practices where movement was incidental, decorative, added temporarily for tourism display, or absent from stable transmission were excluded.

Eligible items were assigned to three analytical categories (Table 2). The categories were allowed to overlap because some practices combine competitive, performative, and ritual functions. Classification was applied to distinct official-list entries before county-level aggregation. Items fitting more than one category retained all applicable codes but were counted once in the total SRICH stock.

**Table 2:** Operational classification of sports-related intangible cultural heritage.

Decision or category	Operational rule	Exclusion boundary	Typical forms
General inclusion	The embodied component is indispensable to the practice, and it displays an identifiable sport-related structure	Incidental movement, decorative performance, tourist-added display, or physical participation without stable transmission	—
S1: Core Traditional Sports	Physical skill, training, competition, game rules, or combat techniques form the central structure	Practices without stable rules, skill transmission, training, or recognizable competitive structure	Martial arts, wrestling, dragon boat racing, traditional competitive games
S2: Embodied Performance Heritage	Codified and skill-dependent bodily movement or coordinated group performance is the principal medium of expression	Dance or performance in which movement is primarily decorative or only accompanies another cultural activity	Dragon and lion performances, drum dances, Baishou dance, Zhanggu dance, Roulianxiang, Maogusi
S3: Sport–Ritual Hybrid Heritage	Physical competition or structured collective performance is integral to the ritual sequence, customary calendar, or communal process	Festivals containing physical activity that is optional, recently added, or unrelated to the ritual structure	Dragon boat customs, ritual martial performances, fire-dragon customs, Maolong, Zhalong, Shaolong
Multiple coding	An item may receive more than one category code when it satisfies each relevant rule	Overlapping category codes must not be added to calculate the total SRICH stock	S1–S3 combinations permitted

Category membership is non-exclusive. A project may satisfy more than one category rule but is counted once in the total number of distinct SRICH projects.

To assess the reproducibility of the classification, two authors independently reviewed the 110 distinct official-list entries using the predefined codebook. S1, S2, and S3 were recorded as separate binary decisions because category membership was non-exclusive. The complete multi-label assignments agreed for 103 of the 110 entries, corresponding to an exact-match agreement rate of 93.6%. Category-specific Cohen’s kappa values were 0.91 for S1, 0.85 for S2, and 0.82 for S3. The seven entries with project-level discrepancies were re-examined against their official descriptions. Disagreements were resolved by discussion according to the constitutive feature of each practice; unresolved cases were adjudicated by the third author. The final coding retained every category supported by the decision rules. The 2024 inventory therefore contained 35 S1 memberships, 69 S2 memberships, and 17 S3 memberships, yielding 121 category memberships across 110 projects. The additional memberships resulted from projects satisfying more than one category rule.

Two borderline cases illustrate the coding procedure. “Dragon Boat Festival customs in the hometown of Qu Yuan, including dragon-boat racing” was retained as S3 rather than being assigned to S1 solely because a race was included in the official description. The entry was treated as a ritual-festival complex in which the customary calendar and ceremonial context organized the practice. By contrast, Dejiang Tujia dragon dance was coded as both S1 and S2 because technical bodily skills and coordinated group performance were both integral to the documented practice. It was not assigned to S3 in the absence of a constitutive ritual obligation. Movements or performances added solely for tourism presentation were not used as evidence for classification.

### 2.3 Data sources and processing

SRICH data were compiled from national, provincial, and county-level representative lists issued by the State Council, the China Intangible Cultural Heritage Network, and the cultural and tourism authorities of Hunan, Hubei, Guizhou, and Chongqing. Project names, recognition batches, declaring areas, and safeguarding units were cross-checked. After deduplication, temporal screening, and spatial matching, 110 distinct SRICH records corresponding to 118 county–project records were retained across 71 county-level units.

Tourism data for 2010, 2015, 2019, and 2024 were obtained from county statistical yearbooks, statistical communiqués, government reports, official tourism publications, and the EPS data platform. Following the scale–performance and input–output frameworks used in comparable heritage–tourism studies [27–29,47], six positive indicators were constructed: tourist arrivals (X1) and tourism revenue (X2) for tourism output; 3A-or-above scenic attractions (X3), star-rated hotels (X4), and total road mileage (X5) for tourism supply; and the ratio of total tourism revenue to county GDP (X6) as a measure of tourism revenue relative to the size of the county economy (Table 3). Indicator definitions, units, county codes, and reporting conventions were harmonized across the four provincial regions. Administrative boundaries were obtained from the Standard Map Service System of the China Ministry of Natural Resources.

**Table 3:** Tourism development indicators and data sources.

Dimension	Code	Indicator	Unit	Conceptual meaning	Principal data sources	Entropy weight
Tourism output	X1	Total tourist arrivals	$\text{1}{\text{0}}^{\text{4}}$ person-times	Tourism market scale	County statistical yearbooks, statistical communiqués, government work reports, and official cultural and tourism publications	0.3197
Tourism output	X2	Total tourism revenue	CNY$\text{1}{\text{0}}^{\text{8}}$	Economic output of tourism	County statistical yearbooks, statistical communiqués, government work reports, and official cultural and tourism publications	0.3019
Supply and supporting capacity	X3	Number of scenic attractions rated 3A or above	Number	Formally recognized tourism-attraction supply	Official lists issued by provincial and prefectural cultural and tourism authorities	0.1026
Supply and supporting capacity	X4	Number of star-rated hotels	Number	Formal tourism reception capacity	Statistical yearbooks, statistical communiqués, and cultural and tourism authorities	0.0789
Supply and supporting capacity	X5	Total road mileage	km	Scale of supporting transport infrastructure	Statistical yearbooks, statistical communiqués, and cultural and tourism authorities	0.1134
Tourism revenue relative to county GDP	X6	Tourism revenue-to-GDP ratio	%	Tourism revenue relative to the size of the county economy	Calculated as total tourism revenue divided by county GDP and multiplied by 100	0.0835

X1 and X2 measure absolute tourism activity, whereas X6 measures the scale of tourism revenue relative to county GDP. Tourism revenue and county GDP were converted to constant 2010 prices using province-specific consumer price indices before X6 was calculated.

X1 and X2 measure the absolute scale of tourism demand and tourism output, whereas X6 measures total tourism revenue relative to county GDP. Although tourism revenue appears in both X2 and the numerator of X6, the two indicators capture different dimensions: absolute tourism revenue and tourism revenue relative to the county economy. X6 was therefore retained as a ratio indicator of tourism revenue relative to county GDP, while the entropy weights were interpreted as data-derived contributions to the composite index rather than as independent measures of causal importance. The overlap between X2 and X6 was treated as a measurement consideration when interpreting the composite tourism index. An additional specification excluding X6 produced a similar temporal pattern and county ranking, further supporting the stability of the main results.

The balanced panel comprised 284 county–year observations and 1,704 indicator cells. Missingness affected 56 primary cells: in 2010, X1 and X2 each had 12 missing observations (16.9%), and X4 had 11 (15.5%); in 2015, X1 and X2 had 5 (7.0%) and 7 (9.9%), respectively; and in 2019, they had 4 (5.6%) and 5 (7.0%), respectively. By province, reported in the order Chongqing/Hunan/Guizhou/Hubei and using 7/37/16/11 counties as denominators, the corresponding counts and rates were: 2010 X1/X2, 2/4/4/2 (28.6/10.8/25.0/18.2%); 2010 X4, 2/4/3/2 (28.6/10.8/18.8/18.2%); 2015 X1, 1/2/1/1 (14.3/5.4/6.3/9.1%); 2015 X2, 1/3/2/1 (14.3/8.1/12.5/9.1%); 2019 X1, 0/2/1/1 (0/5.4/6.3/9.1%); and 2019 X2, 1/2/1/1 (14.3/5.4/6.3/9.1%). The 24 missing X2 observations affected the derived X6 values, which were recalculated after completing X2 and county GDP.

Internal gaps were filled by piecewise linear interpolation only when original observations were available on both sides of the missing year. A missing 2015 value was interpolated between 2010 and 2019, and a missing 2019 value between 2015 and 2024. If either bracketing observation was missing, no imputed value was used as an interpolation anchor. The longest continuous missing run was three benchmark observations; these endpoint or consecutive gaps were handled using multiple imputation by chained equations with predictive mean matching. Twenty completed datasets were generated using five observed donor values per missing cell, and H Q, D, and the spatial statistics were recalculated for each dataset. No endpoint extrapolation was used. Missing values at the beginning or end of the observation sequence were handled only through the multiple-imputation procedure described above, and no extrapolated value was used as an interpolation anchor. The pooled regional mean D was 0.1785 in 2010 and 0.4071 in 2024, with 95% between-imputation ranges of 0.1774–0.1796 and 0.4069–0.4073. The estimated increase in mean D was 0.2285, the largest annual difference was 0.0064, and year-specific county-ranking correlations ranged from Spearman’s (ρ=0.9965) to 0.9992.

As a lower-tail sensitivity check, replacing the 2010 Q values of the 12 lowest-Q counties with province-specific fifth-percentile values changed mean Q from 0.111808 to 0.111150 (−0.59%) and mean D from 0.192173 to 0.191729 (−0.23%). Nominal tourism revenue and county GDP were converted to constant 2010 prices using the corresponding province-specific annual consumer price index, with 2010 set equal to 100. X6 was then calculated as constant-price tourism revenue divided by constant-price county GDP and multiplied by 100.

### 2.4 Research methods

A stepwise analytical framework was developed to examine SRICH from the county-level distribution of officially recognized project records to its relationship with tourism development. First, cumulative counts, the geographical concentration index, kernel density estimation, and the standard deviational ellipse were used to identify changes in the scale, county-level concentration, smoothed density cores, and directional tendency of the official records across the four benchmark years. Second, SRICH endowment and tourism development were converted into comparable county-level indices, providing a consistent basis for evaluating their relationship. The coupling coordination model was then applied to assess whether the two systems developed in a coordinated manner, while the relative-balance index identified which system lagged. Finally, global and local Moran’s (I) were used to determine whether SRICH endowment and coupling coordination formed significant spatial clusters. This combination of record-based and county-level methods links the administrative geography of heritage recognition with system evaluation, with each method addressing a distinct but connected aspect of the research questions. Fig 2 summarizes this workflow.

**Fig 2:**
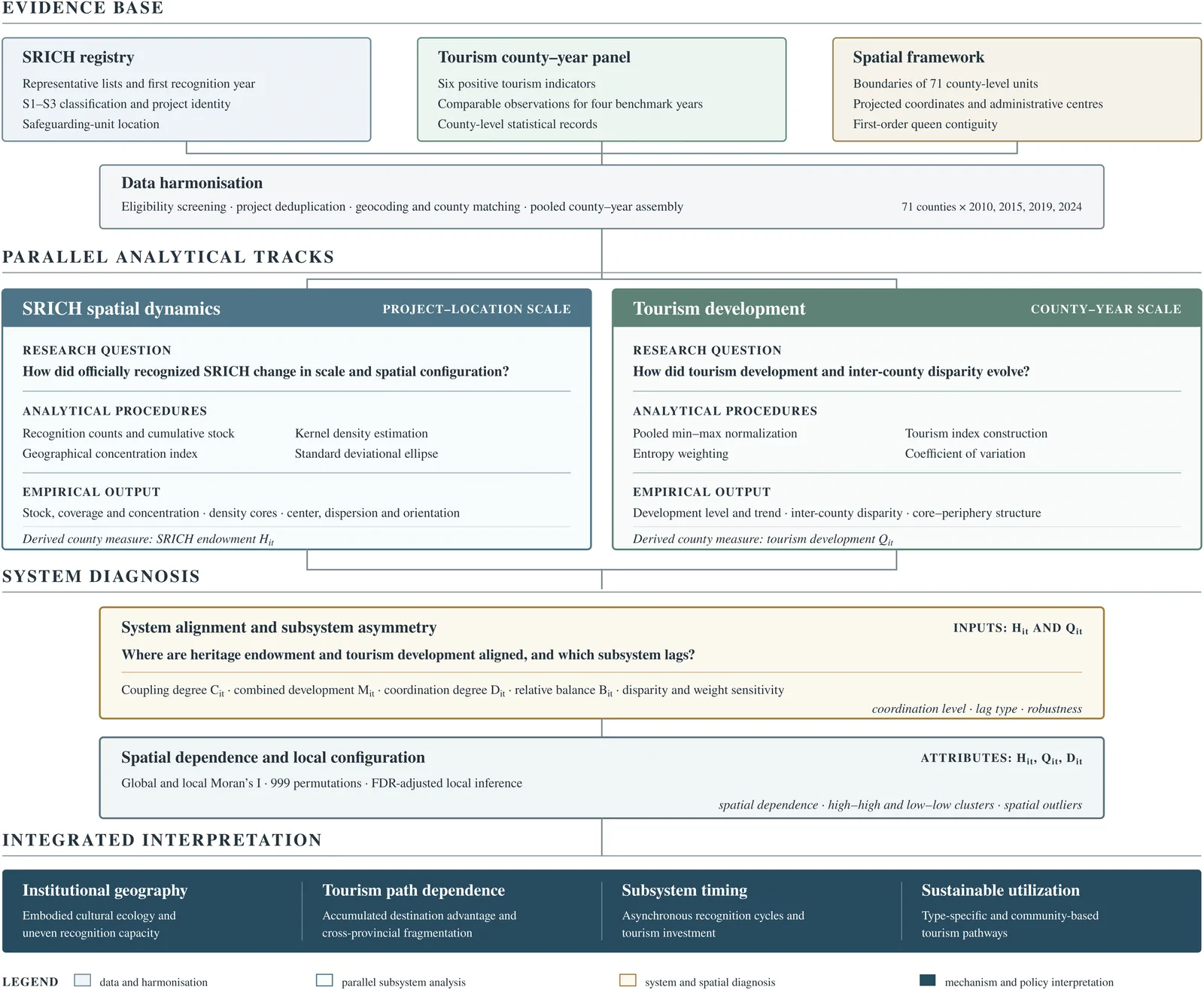
Analytical framework of the study.

#### 2.4.1 Geographical concentration index.

The geographical concentration index measures the degree to which a spatial phenomenon is unevenly distributed among geographical units [22,48]. Whereas the cumulative number of SRICH projects reflects changes in resource scale, the concentration index indicates whether these projects are concentrated in a limited number of counties or distributed more evenly across the study area. In this study, the index was calculated for 2010, 2015, 2019, and 2024 to examine whether the expansion of officially recognized SRICH was accompanied by increasing geographical concentration or a wider county-level distribution of officially recognized records.

The geographical concentration index was calculated as:


$$\begin{array}{c} G_{t}=\text{100}\sqrt{\sum_{i=\text{1}}^{m}{\left(\frac{n_{it}}{N_{t}}\right)}^{\text{2}}},\;N_{t}=\sum_{i=\text{1}}^{m}n_{it} \end{array}$$ (1)

where ${\text{G}}_{\text{t}}$ is the geographical concentration index in year t;${\text{n}}_{\text{it}}$ is the cumulative number of eligible SRICH projects associated with county i by year t; ${\text{N}}_{\text{t}}$ is the corresponding total across all counties; and m = 71 is the number of county-level units. A project officially associated with more than one county was counted once in each relevant county; consequently,${\text{N}}_{\text{t}}$ represents the total number of county–project records rather than the region-wide number of distinct projects.

For a fixed set of m counties,${\text{G}}_{\text{t}}$ ranges from $(\text{100}/\sqrt{\text{m}})$ to 100. Under a perfectly equal distribution across the 71 counties, the reference value is:${\text{G}}_{\text{0}}=\frac{\text{100}}{\sqrt{\text{71}}}=\text{11}.\text{87}$.

A value closer to 100 indicates stronger geographical concentration, whereas a value closer to ${\text{G}}_{\text{0}}$ indicates a more even distribution. Accordingly, an increase in ${\text{G}}_{\text{t}}$ over time denotes greater concentration, while a decrease indicates that officially recognized SRICH records are distributed more evenly across counties [48].

#### 2.4.2 Kernel density estimation.

Kernel density estimation (KDE) converts discrete point records into a continuous density surface by assigning greater weights to nearby observations [49,50]. Because the official inventories associate projects with counties but do not provide consistent practice-site coordinates, each county–project record was located at the administrative center of the corresponding county. A project associated with several counties generated one record for each county, while distinct projects assigned to the same center remained separate observations. The cumulative samples contained 52, 80, 94, and 118 project–location records in 2010, 2015, 2019, and 2024, respectively. KDE was used to identify the location and spatial extent of concentrations in the administrative geography of official SRICH recognition.

The smoothed density of county-represented SRICH records at location s in year t was estimated using the quartic kernel:


$$\begin{array}{c} {\hat{\lambda }}_{t}\left(s\right)=\sum_{k=\text{1}}^{n_{t}}\frac{\text{3}}{\pi h^{\text{2}}}{\left[\text{1}-{\left(\frac{d_{ks}}{h}\right)}^{\text{2}}\right]}^{\text{2}}\text{1}\left(d_{ks}<h\right) \end{array}$$ (2)

where $\hat{\lambda }\text{t}(\text{s}$) is the estimated density of county-represented official SRICH records at location s in year t; ${\text{n}}_{\text{t}}$ is the cumulative number of eligible project–location records by year t; dks is the distance between the county-center point assigned to record k and location s; h is the bandwidth controlling the spatial range of the density estimate; and $\text{1}({\text{d}}_{\text{ks}}<\text{h}$) is an indicator function that limits the contribution of record k to locations within the bandwidth.

All records were equally weighted, and planar distances were calculated in WGS 84/UTM zone 49N (EPSG:32649). A bandwidth of 55.247523 km, derived from the complete 2024 sample using the ArcGIS default procedure, was applied to all four years. The raster cell size was fixed at 2 km, and the output extent and density units were held constant. Density values were reported as project–location records per 10,000 km². For mapping, the four surfaces were divided into five levels—very low, low, moderate, high, and very high—using identical break values. Annual peak densities were extracted from the continuous surfaces.

Because the annual samples were cumulative, rising density values partly reflect the expansion of official-list coverage. KDE therefore indicates where recorded concentration cores formed and expanded, whereas temporal changes in relative concentration were evaluated using the geographical concentration index. The resulting surfaces represent the smoothed administrative distribution of official records; they do not locate exact practice sites, delineate cultural territories, or measure the vitality of living transmission.

#### 2.4.3 Standard deviational ellipse.

The standard deviational ellipse (SDE) is a spatial statistical method that summarizes the central tendency, dispersion, and directional orientation of a point distribution [51]. By fitting an ellipse to the spatial arrangement of observations, it identifies the mean center, spatial spread along the two principal axes, and dominant orientation of the distribution. In this study, SDE was fitted to the county-center points assigned to officially recognized SRICH records in each benchmark year. Its parameters therefore summarize the regional administrative geography of official recognition rather than the precise locations or movement of practitioner communities.

For each benchmark year, the mean center was calculated as:


$$\begin{array}{c} {\overset{―}{\text{x}}}_{\text{t}}=\frac{\text{1}}{{\text{n}}_{\text{t}}}\sum_{\text{k}=\text{1}}^{{\text{n}}_{\text{t}}}{\text{x}}_{\text{kt}},\;\;{\overset{―}{\text{y}}}_{\text{t}}=\frac{\text{1}}{{\text{n}}_{\text{t}}}\sum_{\text{k}=\text{1}}^{{\text{n}}_{\text{t}}}{\text{y}}_{\text{kt}} \end{array}$$ (3)

where ${\text{x}}_{\text{kt}}$ and ${\text{y}}_{\text{kt}}$ are the projected coordinates of the county administrative-center point assigned to project record (k) in year (t). the spatial covariance matrix is:


$$\begin{array}{c} \Sigma_{\text{t}}=\frac{\text{1}}{{\text{n}}_{\text{t}}}(\begin{array}{cc} \sum_{\text{k}=\text{1}}^{{\text{n}}_{\text{t}}}{\tilde{\text{x}}}_{\text{kt}}^{\text{2}} & \sum_{\text{k}=\text{1}}^{{\text{n}}_{\text{t}}}{\tilde{\text{x}}}_{\text{kt}}{\tilde{\text{y}}}_{\text{kt}}\\ \sum_{\text{k}=\text{1}}^{{\text{n}}_{\text{t}}}{\tilde{\text{x}}}_{\text{kt}}{\tilde{\text{y}}}_{\text{kt}} & \sum_{\text{k}=\text{1}}^{{\text{n}}_{\text{t}}}{\tilde{\text{y}}}_{\text{kt}}^{\text{2}} \end{array}) \end{array}$$ (4)

The eigenvalues of $\Sigma_{\text{t}}$ determine dispersion along the major and minor axes, while the eigenvector associated with the larger eigenvalue determines the orientation of the ellipse. An unweighted one-standard-deviation ellipse was generated for each benchmark year. Changes in the mean center, major and minor axes, ellipse area, and azimuth were used to describe shifts in the spatial configuration of county-represented official records. All distance-based operations were performed in a meter-based projected coordinate system to avoid distortions associated with geographic coordinates. These changes should not be interpreted as evidence that the cultural practices themselves moved or expanded in the same direction.

#### 2.4.4 Entropy weight method.

The entropy weight method assigns higher weights to indicators showing greater variation across the observed sample [52] and has been applied to construct weighted composite indices for coupling coordination analysis of ICH and tourism development [53]. In this study, entropy weighting was applied only to the six tourism indicators. SRICH endowment was measured directly from the cumulative stock of distinct eligible projects.

The SRICH endowment index was normalized over the complete county–year panel as:


$$\begin{array}{c} H_{it}=\frac{h_{it}-h_{min}}{h_{max}-h_{min}},\;h_{min}=\underset{i,t}{min}\left(h_{it}\right),\;h_{max}=\underset{i,t}{max}\left(h_{it}\right) \end{array}$$ (5)

where ${\text{h}}_{\text{it}}$ is the cumulative number of distinct eligible SRICH projects in county i by year t, and ${\text{H}}_{\text{it}}\in [\text{0},\text{1}]$. A project assigned to more than one of S1–S3 was counted once in the overall stock, while a project officially associated with several counties contributed once to each relevant county. The index measures formal SRICH endowment and does not capture transmission vitality, safeguarding quality, or tourism utilization.

The tourism development index was constructed from the six positive indicators listed in Table 3. All 284 county–year observations (m=71, K=4) were pooled to obtain a common set of normalization parameters and entropy weights. Indicator j was standardized as:


$$\begin{array}{c} z_{ijt}=\frac{x_{ijt}-x_{j,min}}{x_{j,max}-x_{j,min}} \end{array}$$ (6)

where ${\text{x}}_{\text{ijt}}$ is the original value, ${\text{z}}_{\text{ijt}}\in [\text{0},\text{1}]$, and ${\text{x}}_{\text{j},\text{min}}$ and ${\text{x}}_{\text{j},\text{max}}$ are the minimum and maximum of indicator j across the full panel.

The proportion of each observation under indicator j was calculated as:


$$\begin{array}{c} p_{ijt}=\frac{z_{ijt}}{\sum_{\tau =\text{1}}^{K}\sum_{r=\text{1}}^{m}z_{rj\tau }} \end{array}$$ (7)

where the summation is over all county–year observations for indicator j.

The entropy value ${\text{e}}_{\text{j}}$ and information utility ${\text{d}}_{\text{j}}$ of indicator j were obtained from:


$$\begin{array}{c} e_{j}=-\frac{\text{1}}{ln\left(mK\right)}\sum_{\tau =\text{1}}^{K}\sum_{r=\text{1}}^{m}p_{rj\tau }{lnp}_{rj\tau },\;d_{j}=\text{1}-e_{j} \end{array}$$ (8)

where ${\text{p}}_{\text{rj}\tau }{\text{ln}\text{p}}_{\text{rj}\tau }$ was defined as zero when ${\text{p}}_{\text{ijt}}=\text{0}$. The entropy weight was calculated as:


$$\begin{array}{c} w_{j}=\frac{d_{j}}{\sum_{l=\text{1}}^{q}d_{l}},\;\;\sum_{j=\text{1}}^{q}w_{j}=\text{1} \end{array}$$ (9)

Finally, the tourism development index was calculated as:


$$\begin{array}{c} Q_{it}=\sum_{j=\text{1}}^{q}w_{j}z_{ijt},\;\;\text{0}\leq Q_{it}\leq \text{1} \end{array}$$ (10)

A higher ${\text{Q}}_{\text{it}}$ indicates a higher composite level of tourism development. Because normalization and weighting were based on the pooled panel, the same measurement scale was applied to all four benchmark years. The resulting weights reflect cross-county and temporal variation within the observed sample; they do not represent normative judgments about the substantive importance of the indicators.

#### 2.4.5 Coupling coordination degree model.

The coupling coordination degree model (CCDM) combines subsystem similarity with their composite development level [54] and has been applied to examine relationships between ICH resources and tourism development [29]. Here, it was used to compare the joint configuration of SRICH endowment and tourism development across counties and benchmark years.

The coupling degree was calculated as:


$$\begin{array}{c} C_{it}=\frac{\text{2}\sqrt{H_{it}Q_{it}}}{H_{it}+Q_{it}},\;\text{0}\leq C_{it}\leq \text{1} \end{array}$$ (11)

where ${\text{H}}_{\text{it}}$ and ${\text{Q}}_{\text{it}}$ are the standardized SRICH endowment and tourism development indices. A higher ${\text{C}}_{\text{it}}$indicates greater similarity between the two scores, although equally low scores can also produce a high value. The combined development index was therefore defined as:


$$\begin{array}{c} M_{it}=\alpha H_{it}+\beta Q_{it},\;\alpha +\beta =\text{1} \end{array}$$ (12)

where α and β denote the subsystem weights. Equal weights were assigned in the baseline analysis α=β=0.5. The coupling coordination degree was calculated as:


$$\begin{array}{c} D_{it}=\sqrt{C_{it}M_{it}},\;\;\text{0}\leq D_{it}\leq \text{1} \end{array}$$ (13)

Under the equal-weight specification, Equations (11)–(13) simplify to ${\text{D}}_{\text{it}}=({\text{H}}_{\text{it}}{\text{Q}}_{\text{it}}{)}^{\text{1}/\text{4}}$. Thus, ${\text{D}}_{\text{it}}$ increases with the joint product of the subsystem scores and equals zero when either score is zero. It does not independently indicate whether their relative balance has improved. When ${\text{H}}_{\text{it}}+{\text{Q}}_{\text{it}}=\text{0}$, ${\text{C}}_{\text{it}}$ and ${\text{D}}_{\text{it}}$ were set to zero [24].

For descriptive mapping, ${\text{D}}_{\text{it}}$ was divided into five equal-width classes (Table 4); continuous values were retained in all numerical and statistical analyses.

**Table 4:** Classification of the coupling coordination degree.

Range	Coordination state
0.00≤D<0.20	Severe imbalance
0.20≤D<0.40	Moderate imbalance
0.40≤D<0.60	Transitional stage
0.60≤D<0.80	Moderate coordination
0.80≤D≤1.00	High coordination

The five categories use equal-width intervals of 0.20 on the [0,1] scale and are intended for descriptive classification only. Continuous D values were retained for all statistical analyses.

Because both subsystem indices are normalized to ([0,1]), the coupling coordination degree D also lies within the unit interval. We therefore divided this interval into five equal-width bands of 0.20, using 0.20, 0.40, 0.60, and 0.80 as descriptive cut-points. This five-band scheme follows the conventional classification used in coupling-coordination studies [24,25,27,29] and provides a transparent descriptive classification of increasing values of D. The thresholds are not treated as universal natural breaks, causal thresholds, or statistical significance criteria. They are used only for descriptive mapping and the composition of coordination states, whereas all temporal, ranking, sensitivity, and spatial analyses use continuous D values.

The direction of subsystem imbalance was identified using the normalized relative-balance index:


$$\begin{array}{c} B_{it}=\frac{H_{it}-Q_{it}}{H_{it}+Q_{it}},\;-\text{1}\leq B_{it}\leq \text{1} \end{array}$$ (14)

A negative value indicates an SRICH-endowment lag, while a positive value indicates a tourism-development lag. Lower values of $\left|{\text{B}}_{\text{it}}\right|$ denote closer similarity between the subsystem scores. If both scores were zero, ${\text{B}}_{\text{it}}$ was set to zero and the county was recorded separately as joint low-level stagnation. The index was used diagnostically and did not enter the calculation of ${\text{D}}_{\text{it}}$.

The equal-weight assumption was tested under α,β=(0.4,0.6) and (0.6,0.4). County rankings, coordination classes, and spatial patterns were compared with the baseline results. This test assesses sensitivity to weight selection; it does not address official-list coverage because ${\text{H}}_{\text{it}}=\text{0}$ fixes ${\text{D}}_{\text{it}}=\text{0}$ under every weighting scenario.

To distinguish changes within previously covered counties from the expansion of official recognition, counties were grouped by their 2010 list status. The baseline-covered cohort $B_{\text{0}}$ comprised the 35 counties with at least one eligible project in 2010. The entrant cohort Ecomprised the 19 counties with no eligible project in 2010 but at least one by 2024; the remaining 17 counties had no eligible project in either endpoint year. The change in the regional mean was decomposed as: ${\overset{―}{\text{D}}}_{\text{2024}}-{\overset{―}{\text{D}}}_{\text{2010}}=\frac{\text{1}}{\text{m}}\sum_{\text{i}\in {\text{B}}_{\text{0}}}\left({\text{D}}_{\text{i},\text{2024}}-{\text{D}}_{\text{i},\text{2010}}\right)+\frac{\text{1}}{\text{m}}\sum_{\text{i}\in \text{E}}{\text{D}}_{\text{i},\text{2024}},\text{m}=\text{71}$. The first term measures change among counties covered in 2010, and the second captures the component associated with post-2010 entrants. A fixed-support regional mean was also calculated as: ${\overset{―}{\text{D}}}_{\text{t}}^{\text{FS}}=\frac{\text{1}}{\text{m}}\sum_{\text{i}\in B_{\text{0}}}{\text{D}}_{\text{it}}=\frac{\text{1}}{\text{71}}\sum_{\text{i}\in B_{\text{0}}}{\text{D}}_{\text{it}}$.

thereby retaining the baseline cohort in the numerator and all 71 counties in the denominator. Within $B_{\text{0}}$, changes in H, Q, C, M, D, and |B| were compared across the four years. Two-sided Wilcoxon signed-rank tests assessed the 2010–2024 changes in C, M, D, and |B|. The decomposition is an accounting identity, not a causal estimate; the entrant component includes official-list entry and the subsystem levels subsequently reached by those counties. It should therefore be interpreted as the share of the observed change associated with changes in county coverage and subsequent recorded subsystem levels, rather than as the causal effect of official listing. Accordingly, D should be read as a comparative joint-level measure, not as direct evidence of causal interaction, functional integration, or sustainable tourism outcomes.

#### 2.4.6 Regional disparity and spatial autocorrelation analysis.

The coefficient of variation CV was used to describe relative disparities in tourism development and the coupling coordination degree across counties [55]. Global and local Moran’s (I) were then applied to H, Q, and D to test overall spatial dependence and identify significant local clusters and spatial outliers [56,57]. Global Moran’s I summarizes neighboring similarity across the region, while local Moran’s I identifies significant clusters and spatial outliers under the specified weight matrix.

For ${\text{Q}}_{\text{it}}$ and ${\text{D}}_{\text{it}}$, the CV was calculated as:


$$\begin{array}{c} CV_{t}=\frac{\sqrt{\frac{\text{1}}{m}\sum_{i=\text{1}}^{m}{\left(v_{it}-{\overset{―}{v}}_{t}\right)}^{\text{2}}}}{{\overset{―}{v}}_{t}} \end{array}$$ (15)

where (m = 71), ${\text{v}}_{\text{it}}$ is the value for county i in year t, and ${\overset{―}{\text{v}}}_{\text{t}}$ is the corresponding county mean. A lower $\text{C}{\text{V}}_{\text{t}}$ indicates less dispersion relative to the regional mean. Because CV is also affected by changes in the mean and the number of zero-value counties, its decline was not treated as independent evidence of intercounty convergence.

Spatial relationships were represented by a row-standardized first-order queen-contiguity matrix. Counties sharing either a boundary or a vertex were defined as neighbors, and ${\text{w}}_{\text{ii}}=\text{0}$ [58]. Global Moran’s (I) was used to assess overall spatial autocorrelation:


$$\begin{array}{c} I_{t}=\frac{m}{S_{\text{0}}}\frac{\sum_{i=\text{1}}^{m}\sum_{j=\text{1}}^{m}w_{ij}\left(v_{it}-{\overset{―}{v}}_{t}\right)\left(v_{jt}-{\overset{―}{v}}_{t}\right)}{\sum_{i=\text{1}}^{m}{\left(v_{it}-{\overset{―}{v}}_{t}\right)}^{\text{2}}},\;S_{\text{0}}=\sum_{i=\text{1}}^{m}\sum_{j=\text{1}}^{m}w_{ij} \end{array}$$ (16)

where ${\text{V}}_{\text{it}}$ successively represents ${\text{H}}_{\text{it}}$, ${\text{Q}}_{\text{it}}$, and ${\text{D}}_{\text{it}}$, and ${\text{w}}_{\text{ij}}$ is the spatial weight between counties i and j. A significant positive ${\text{I}}_{\text{t}}$ indicates that counties with similar values tend to be adjacent, while a significant negative value indicates spatial association between dissimilar values. A nonsignificant result provides no evidence against the null hypothesis of spatial randomness.

Local Moran’s (I) was used to locate spatial clusters and outliers:


$$\begin{array}{c} I_{it}=\frac{v_{it}-{\overset{―}{v}}_{t}}{s_{t}^{\text{2}}}\sum_{j=\text{1}}^{m}w_{ij}\left(v_{jt}-{\overset{―}{v}}_{t}\right),\;s_{t}^{\text{2}}=\frac{\text{1}}{m}\sum_{i=\text{1}}^{m}{\left(v_{it}-{\overset{―}{v}}_{t}\right)}^{\text{2}} \end{array}$$ (17)

Significant high–high and low–low observations were interpreted as local clusters, whereas high–low and low–high observations were treated as spatial outliers. Statistical inference for global and local Moran’s (I) was based on 999 random permutations and the resulting pseudo-(p) values. To control for multiple testing, the Benjamini–Hochberg procedure was applied separately to the 71 local tests for each variable–year combination, with (${\text{q}}_{\text{FDR}}=\text{0}.\text{05}$) [59]. Only local observations remaining significant after this adjustment were reported as statistically significant clusters or outliers.

Descriptive statistics, subsystem indices, coupling measures, and CVs were calculated in Microsoft Excel. KDE and SDE analyses were conducted in ArcGIS, while global and local Moran’s (I), Moran scatterplots, and local cluster maps were produced in GeoDa [58].

## 3. Result analysis

### 3.1 Spatiotemporal evolution and spatial structure of SRICH

#### 3.1.1 Overall and category-specific recognition dynamics.

The cumulative stock of officially recognized SRICH increased from 45 projects in 2010–72 in 2015, 86 in 2019, and 110 in 2024, an overall increase of 65 projects (144.4%) (Fig 3). Most of the initial stock was established during the early expansion of the heritage inventory: 42 of the 45 projects recorded by 2010 were recognized between 2006 and 2009. Annual additions averaged 5.40 projects in 2011–2015, declined to 3.50 in 2016–2019, and rose to 4.80 in 2020–2024. The recognition process thus shifted from rapid inventory building to slower and more episodic expansion.

**Fig 3:**
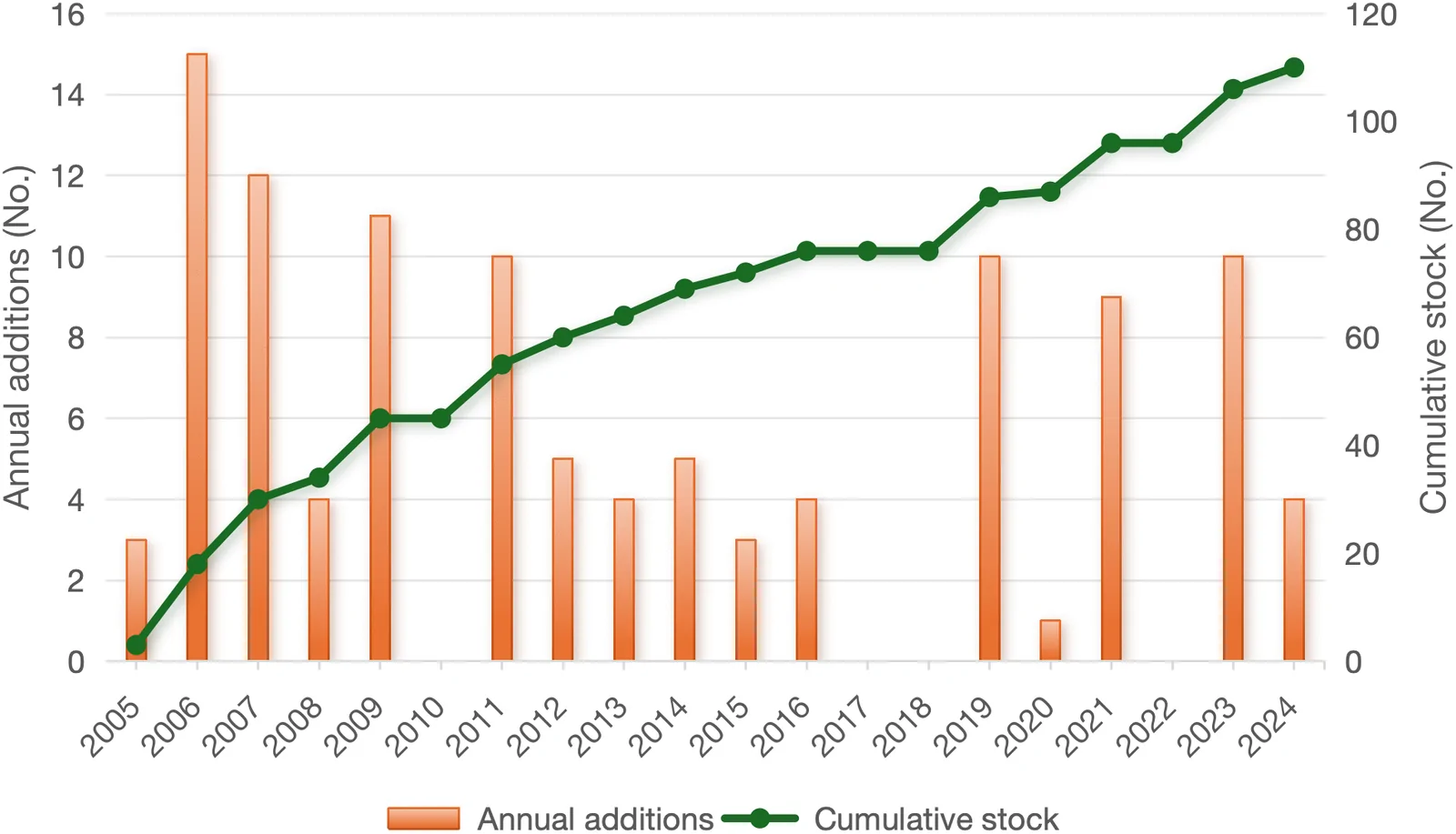
Annual additions and cumulative stock of officially recognized SRICH in the Wuling Mountain Area, 2005–2024. Note: Annual additions are grouped by the year of official recognition, and the cumulative stock represents the number of distinct officially recognized SRICH projects recorded by each year.

Recent additions were strongly concentrated by administrative level and province. Hunan accounted for 19 of the 24 projects added during 2020–2024 (79.2%), and 23 entered through provincial-level lists. No additions were recorded in 2017, 2018, or 2022. Additions were uneven across years and provinces, with recent inventory growth concentrated in Hunan and provincial-level lists.

Category-specific trajectories show that S2 remained dominant while the inventory became more diverse (Fig 4). Embodied performance heritage (S2) increased from 34 projects in 2010–69 in 2024, representing the largest absolute gain (+35). Core traditional sports (S1) rose from 9 to 35 projects, and sport–ritual hybrid heritage (S3) increased from 7 to 17. S1 recorded the fastest proportional growth (288.9%), partly because of its smaller initial stock. Meanwhile, the proportion of distinct projects assigned to S2 declined from 75.6% to 62.7%. Recognition therefore expanded from a predominantly performance-oriented inventory toward a broader combination of competitive, martial, performative, and ritual practices. S2 therefore remained the largest category despite its declining share, while the faster proportional growth of S1 broadened the composition of the recognized inventory.

**Fig 4:**
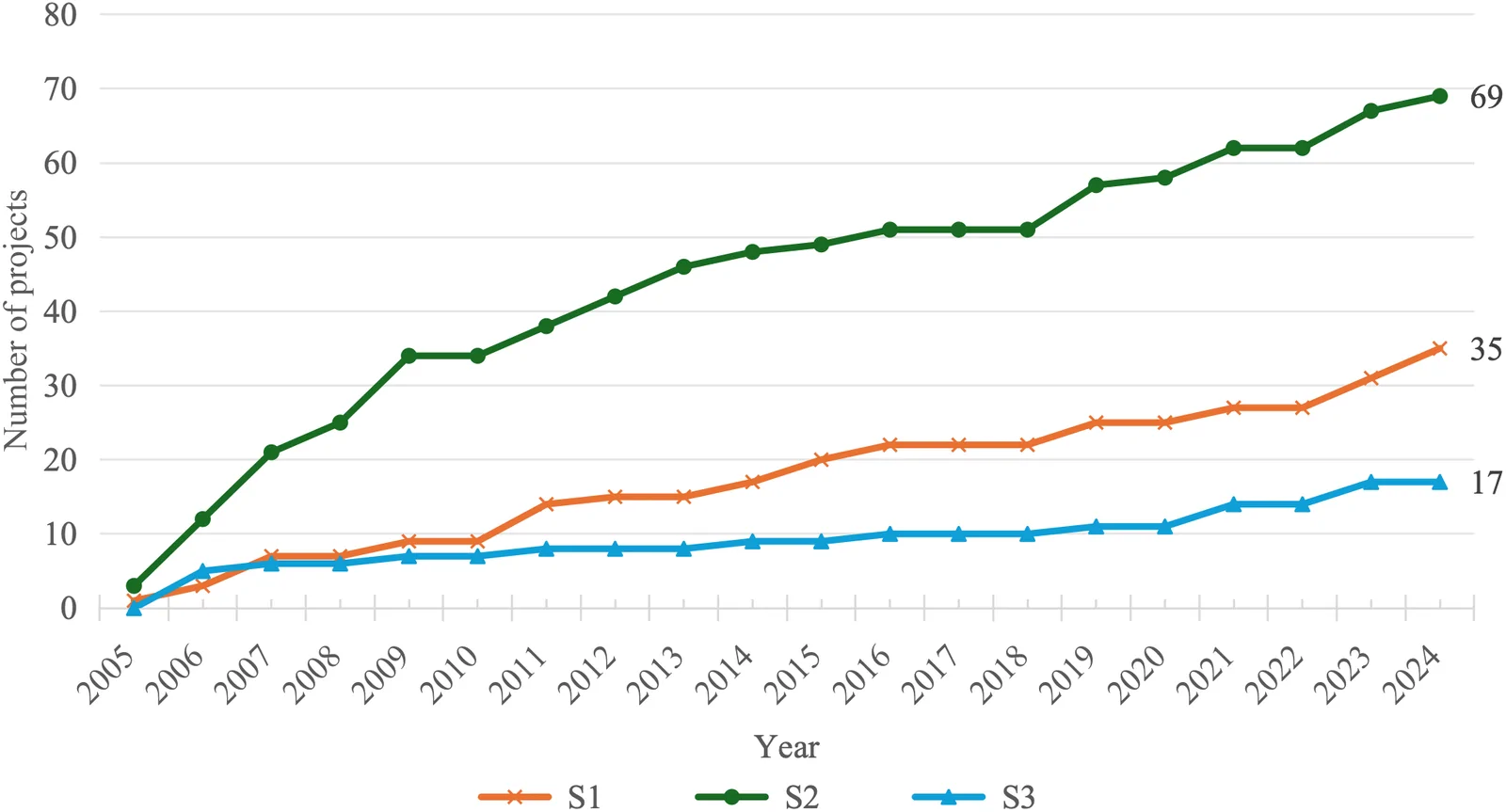
Cumulative evolution of SRICH by analytical category in the Wuling Mountain Area, 2005–2024. Note: Category membership is non-exclusive. A project may be assigned to more than one analytical category but is counted once in the total number of distinct SRICH projects.

Category membership was non-exclusive. In 2024, 11 of the 110 distinct projects satisfied two category definitions, yielding 121 category memberships: eight combined S1 and S2 attributes, one combined S1 and S3, and two combined S2 and S3. These overlaps indicate that athletic technique, performative movement, ritual meaning, and communal participation frequently coexist within the same cultural tradition, supporting the use of a non-exclusive classification.

#### 3.1.2 County coverage and geographical concentration.

The geographical coverage of officially recognized SRICH expanded throughout the study period (Table 5). The number of counties with at least one project increased from 35 in 2010–54 in 2024, raising the coverage rate from 49.3% to 76.1%. The largest increase occurred during 2010–2015, when ten counties entered the inventory, followed by four in 2015–2019 and five in 2019–2024. County project records more than doubled from 52 to 118, while the average number of records per covered county rose from 1.49 to 2.19. Growth therefore occurred through both the entry of previously unrepresented counties and the accumulation of additional projects within covered counties. In 2024, the difference between 118 county records and 110 distinct projects resulted from multi-county associations and remained small relative to the total project stock.

**Table 5:** County coverage and geographical concentration of SRICH.

Year	County project records $(N_{t}$)	Counties with SRICH	Coverage rate (%)	Geographical concentration index (${𝐆}_{𝐭})$	Top-five county share (%)
2010	52	35	49.3	18.64	26.9
2015	80	45	63.4	17.59	26.3
2019	94	49	69.0	16.89	23.4
2024	118	54	76.1	16.12	22.0

The geographical concentration index declined from 18.64 in 2010 to 16.12 in 2024, a decrease of 13.5%. The combined share of the five leading counties also fell from 26.9% to 22.0%. Both measures indicate a gradual spatial diffusion of official recognition. However, the concentration index remained above the uniform-distribution reference of 11.87 in all four years. Huayuan recorded seven county project records in 2024, while Pengshui, Songtao, and Xianfeng each recorded five. The regional inventory thus widened geographically while retaining several established centers of heritage accumulation.

The profile of these leading counties is closely related to the cultural geography of the Wuling Mountain Area. Huayuan, Pengshui, Songtao, and Xianfeng remained prominent centers of officially recognized SRICH. Their position within a widening inventory indicates that broader county coverage coexisted with continued accumulation in established centers. The decline in the top-five county share shows that recognition became less concentrated among the leading counties, even as several retained relatively large project stocks. This distinction between wider coverage and persistent local concentrations provides the spatial context for the subsequent comparison with tourism development.

#### 3.1.3 Evolution of smoothed density cores and administrative spatial configuration.

The KDE surfaces reveal progressive polycentric intensification in the county-based distribution of official SRICH records (Fig 5). In 2010, the highest densities occurred around Huayuan and Xuan’en, reaching 13.73 and 13.16 records per 10,000 km², with smaller nodes near Yanhe and Pengshui. By 2015, the primary peak had shifted to Xianfeng (21.88), accompanied by concentrations around Baojing (20.97) and Yanhe (19.65). Xianfeng remained the largest core in 2019, while the Pengshui–Shizhu cluster became more prominent. In 2024, Huayuan emerged as the dominant core with a density of 33.73, and secondary concentrations persisted around Xianfeng, Yanhe, Pengshui–Shizhu, and Sinan. Huayuan’s renewed prominence coincided with Hunan accounting for 19 of the 24 projects added during 2020–2024.

**Fig 5:**
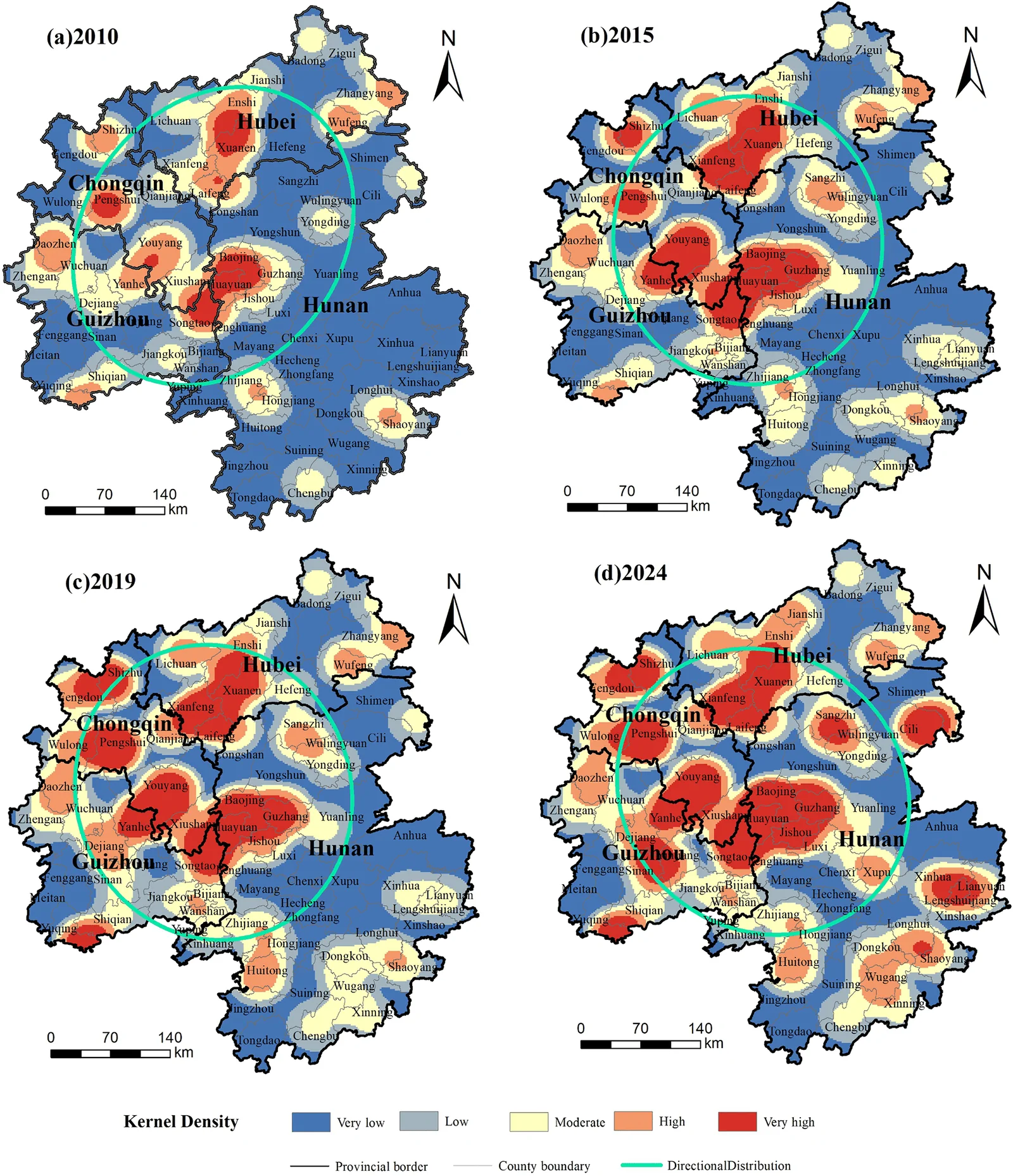
Kernel density surfaces and unweighted one-standard-deviation ellipses of county-represented officially recognized SRICH records, 2010–2024. Note: Each county–project record is located at the administrative center of the corresponding county. The annual samples are cumulative, and a project associated with several counties contributes one record for each county. Administrative boundary data were obtained from the Standard Map Service System of the China Ministry of Natural Resources (http://bzdt.ch.mnr.gov.cn/), under the terms stated on the service website. The maps were created and edited by the authors. The administrative boundary data correspond to map review number GS(2024)0650. No Google Maps, Google Earth, Street View, satellite imagery, or other proprietary basemap was used.

Under the fixed KDE settings, the maximum density increased by 145.6% between 2010 and 2024. Read together with the declining geographical concentration index and top-five county share, this increase indicates polycentric densification: established nodes accumulated additional records while recognition extended to secondary centers and previously uncovered counties. The recurring cores form a cross-provincial belt spanning western Hunan, southwestern Hubei, southeastern Chongqing, and northeastern Guizhou. This configuration corresponds closely to contiguous Miao and Tujia cultural areas, where related festival, martial, ritual, and performance traditions extend across contemporary administrative boundaries. Changes in the dominant peak accompanied shifts in the geographical distribution of newly recorded projects, including the concentration of recent additions in Hunan.

The mean center remained within the central cross-provincial zone, although its position fluctuated between benchmark years (Table 6). It moved 12.47 km southeastward during 2010–2015, 15.08 km southwestward during 2015–2019, and 20.83 km southeastward during 2019–2024. The cumulative inter-period movement was 48.38 km, while the net displacement over the full period was 28.58 km toward the southeast. Relative to a semi-major axis of approximately 170–180 km, this displacement was modest. The modest net displacement and alternating inter-period movements indicate redistribution within the existing regional configuration. The final southeastward shift coincided with Hunan’s large share of recent additions.

**Table 6:** Parameters of the standard deviational ellipses of county-represented officially recognized SRICH records, 2010–2024.

Year	Records (n)	Mean-center longitude (°E)	Mean-center latitude (°N)	Displacement from preceding benchmark (km)	Direction	Semi-major axis (km)	Semi-minor axis (km)	Ellipse area (10⁴ km²)	Axis ratio	Azimuth (°)	Dominant orientation
2010	52	109.2828	28.9766	—	—	180.12	137.31	7.769	1.312	23.46	NNE–SSW
2015	80	109.3691	28.8936	12.47	SE	168.62	138.8	7.352	1.215	177.33	N–S
2019	94	109.2303	28.8339	15.08	SW	173.24	142.48	7.754	1.216	172.79	N–S
2024	118	109.4207	28.7491	20.83	SE	170.22	147.76	7.901	1.152	159.31	NNW–SSE

Each county–project record was located at the administrative center of the corresponding county.

The ellipse area decreased from 7.769 × 10⁴ km² in 2010 to 7.352 × 10⁴ km² in 2015, then recovered to 7.754 × 10⁴ km² in 2019 and 7.901 × 10⁴ km² in 2024. The final area was only 1.70% larger than its 2010 value, despite the increase in county project records from 52 to 118. Over the same period, the semi-major axis contracted from 180.12 to 170.22 km, whereas the semi-minor axis expanded from 137.31 to 147.76 km. The axis ratio consequently declined from 1.312 to 1.152, indicating a more rounded distribution and weaker directional anisotropy. Its dominant orientation changed from NNE–SSW in 2010 to approximately N–S in 2015 and 2019, before rotating to NNW–SSE in 2024.

The KDE and SDE results together identify an increasingly dense network of county-level recognition centers within a largely stable regional envelope. This pattern reflects internal spatial rebalancing and infilling rather than large-scale outward expansion. Together, these results describe increasing record density and modest spatial redistribution within the administrative geography of official recognition.

### 3.2 Spatiotemporal characteristics of tourism development

#### 3.2.1 Temporal evolution and intercounty disparities.

County-level tourism development increased substantially between 2010 and 2024, although growth remained uneven (Table 7 and Fig 6). The regional mean of Q rose from 0.1118 to 0.2892, an increase of 158.7%, while the median increased by 154.3%, from 0.0928 to 0.2360. The mean exceeded the median in every benchmark year, consistent with a right-skewed distribution in which a limited number of high-performing counties raised the regional average. The mean–median gap widened from 0.0190 in 2010 to 0.0585 in 2019 before narrowing slightly to 0.0532 in 2024.

**Table 7:** Temporal evolution and intercounty disparities in tourism development.

Year	N	Mean𝐐	Median𝐐	SD	CV	Minimum	Maximum	Change in mean 𝐐 (%)
2010	71	0.1118	0.0928	0.0573	0.5125	0.0350	0.3547	—
2015	71	0.1727	0.1366	0.0977	0.5660	0.0535	0.5367	54.5
2019	71	0.2713	0.2128	0.1529	0.5636	0.1102	0.7902	57.1
2024	71	0.2892	0.2360	0.1456	0.5034	0.1098	0.7400	6.6

**Fig 6:**
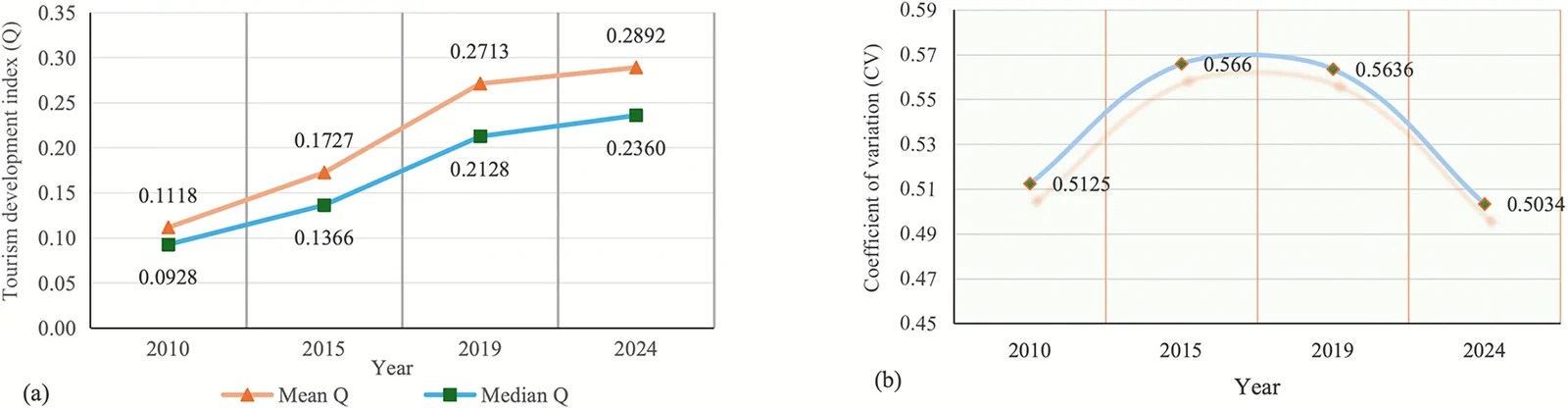
Temporal evolution of county-level tourism development and intercounty disparities in the Wuling Mountain Area, 2010–2024. Note: (a) Regional mean and county median of the tourism development index; (b) coefficient of variation across the 71 counties.

Tourist arrivals and tourism revenue together accounted for 62.16% of the entropy weights, compared with 10.26% for scenic attractions rated 3A or above and 11.34% for total road mileage. The composite index therefore placed greater weight on recorded tourism activity than on any individual supply indicator. X2 captures absolute tourism revenue, whereas X6 captures tourism revenue relative to county GDP; their joint inclusion combines market scale with the relative position of tourism revenue within the county economy. County differences in Q should be interpreted with this composition in mind: higher scores give particular prominence to destinations with larger visitor and revenue totals, alongside their measured tourism supply and road provision.

Most of the regional increase occurred before 2019. Mean Q rose by 54.5% during 2010–2015 and by 57.1% during 2015–2019, compared with 6.6% during 2019–2024. The benchmark comparisons indicate substantially faster growth before 2019 and a smaller net increase during 2019–2024. The latter interval combines changes occurring across several years, while the contrast among periods establishes a clear slowdown in the growth of the composite tourism index.

Intercounty disparities followed an inverted-U trajectory. The CV increased from 0.5125 in 2010 to 0.5660 in 2015 and remained similar in 2019 (0.5636), indicating that established destinations captured a disproportionate share of early growth. By 2024, the CV had fallen by 10.7% to 0.5034, while the standard deviation declined from 0.1529 to 0.1456. During the same period, the median increased by 10.9% and the maximum decreased by 6.4%, suggesting broader improvement among middle-ranking counties and some compression at the upper end. The narrowing remained partial: county values ranged from 0.1098 to 0.7400 in 2024, and only 24 of the 71 counties exceeded the regional mean. The simultaneous decline in CV and standard deviation, together with an increasing median and a lower maximum, indicates reduced dispersion after 2019. Nevertheless, the wide range of county scores and the small number exceeding the regional mean show that this narrowing coexisted with substantial remaining differences.

#### 3.2.2 Spatial differentiation of tourism development.

Fig 7 reveals a persistent spatial hierarchy that gradually evolved from a dominant core–periphery pattern into a more extensive, multi-nodal network. Mean tourism development remained highest in the Chongqing section of the study area, followed by Hubei, Hunan, and Guizhou throughout the four benchmark years. Southeastern Chongqing formed the principal core, with secondary centers emerging around Enshi–Lichuan in southwestern Hubei and Zhangjiajie in northwestern Hunan. The repeated prominence of these centers indicates persistence in the relative spatial hierarchy, even as more counties entered higher tourism-development intervals.

**Fig 7:**
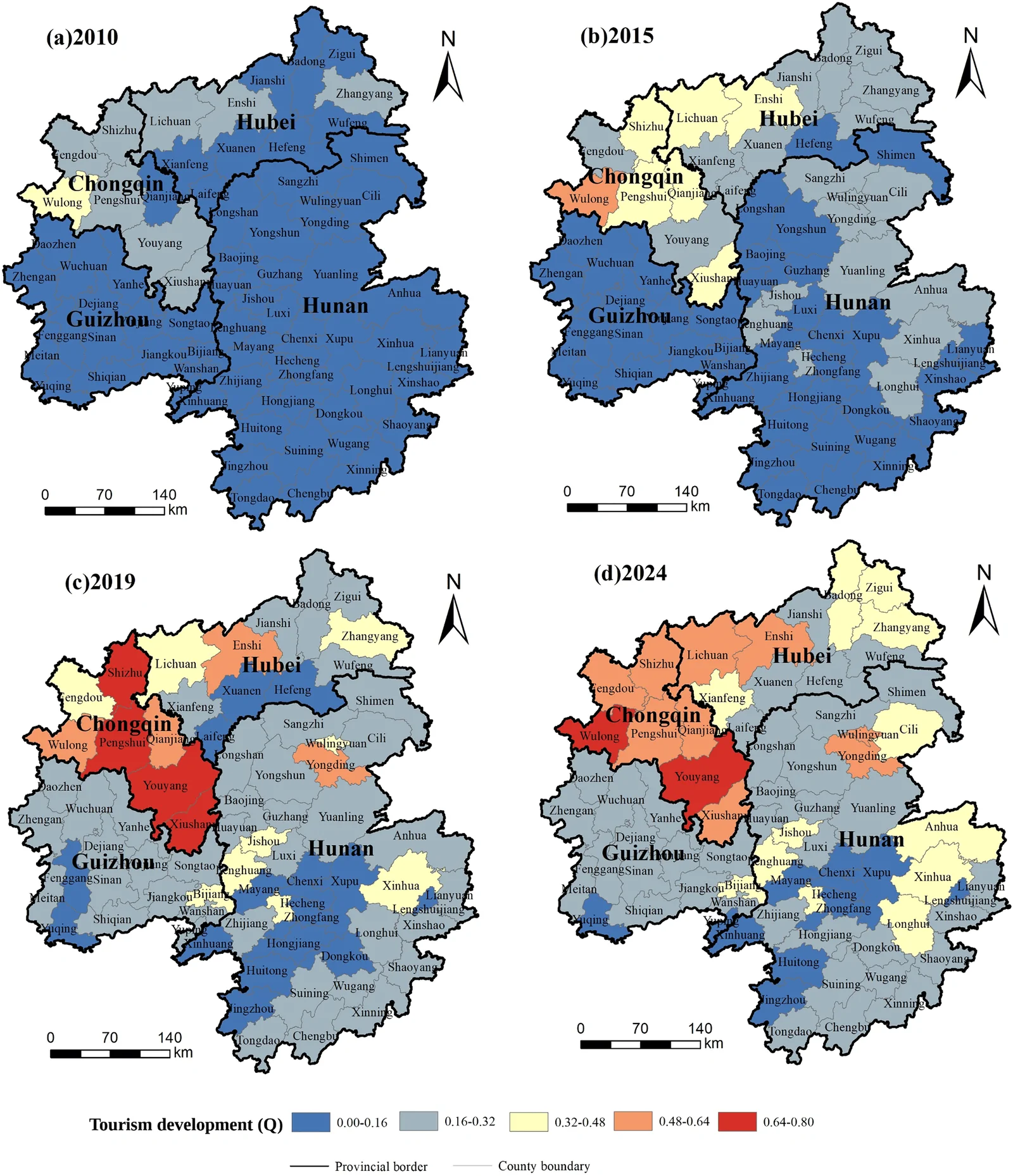
Spatial evolution of county-level tourism development in the Wuling Mountain Area, 2010–2024. Note: Administrative boundary data were obtained from the Standard Map Service System of the China Ministry of Natural Resources (http://bzdt.ch.mnr.gov.cn/), under the terms stated on the service website. The maps were created and edited by the authors. The administrative boundary data correspond to map review number GS(2024)0650. No Google Maps, Google Earth, Street View, satellite imagery, or other proprietary basemap was used.

The spatial reach of tourism development nevertheless expanded substantially. In 2010, 62 counties (87.3%) were in the lowest interval, and Wulong was the only county in a class beginning at 0.32. A relatively developed cluster had already formed across Wulong, Youyang, Pengshui, Fengdu, Shizhu, and Xiushan, accompanied by a smaller Enshi–Lichuan–Changyang node. By 2015, the number of counties in the lowest interval had fallen to 43, while seven counties entered classes of 0.32 or above. The southeastern Chongqing core extended toward Enshi–Lichuan, and Yongding emerged as Hunan’s leading center. This tendency strengthened after 2015: the number of counties reaching 0.32 or above increased to 17 in 2019 and 23 in 2024, equivalent to 23.9% and 32.4% of the regional total. New nodes developed around Wulingyuan, Fenghuang, Jishou, Xinhua, and Hecheng, increasing the spatial complexity of the network.

Wulong, Pengshui, Shizhu, Xiushan, and Enshi remained in the regional top decile at every benchmark, indicating persistence among the leading destinations. This stability coexisted with considerable variation within prefecture-level jurisdictions. In Huaihua, Hecheng had a relatively high Q value, while seven of the ten lowest-ranked counties in 2024 belonged to the same prefecture. The contrast shows that a relatively developed prefectural center can coexist with numerous low-ranking counties within its administrative jurisdiction. By 2024, higher tourism-development values occurred across a wider set of locations, while enduring leading destinations and substantial within-prefecture differences remained evident.

### 3.3 Coupling coordination between SRICH and tourism development

#### 3.3.1 Temporal change, coverage decomposition, and intercounty disparities in coupling coordination.

The regional mean D increased from 0.1922 in 2010 to 0.4029 in 2024, while the median rose from 0 to 0.4574, which falls within the descriptive transitional interval defined in Table 4 (Table 8). Growth was strongest during 2010–2015 and slowed thereafter. Official-list coverage expanded from 35 to 54 counties, reducing the number with H=0 from 36 to 17. Of the 0.2108 increase in mean D, changes within the 35 baseline-covered counties contributed 0.0797 (37.8%), and the 19 post-2010 entrants contributed 0.1310 (62.2%). Holding the original coverage constant produced a 2024 mean of only 0.2719. The entrant component accounted for the larger share of the increase in regional mean D, highlighting the importance of wider official-list coverage and the subsystem scores subsequently recorded in those counties.

**Table 8:** Temporal evolution and coverage decomposition of coupling coordination, 2010–2024.

Table 8−1. Full-sample distribution and coordination-state composition.											
Year	MeanD	MedianD	SDP	CV	Minimum	Maximum	Severe imbalance, n (%)	Moderate imbalance, n (%)	Transitional stage, n (%)	Moderate coordination, n (%)	High coordination, n (%)
2010	0.1922	0.0000	0.2002	1.0416	0.0000	0.5679	36(50.7%)	22(31.0%)	13(18.3%)	0(0.0%)	0(0.0%)
2015	0.2849	0.3688	0.2288	0.8029	0.0000	0.6468	26(36.6%)	18(25.4%)	23(32.4%)	4(5.6%)	0(0.0%)
2019	0.3493	0.4174	0.2532	0.7248	0.0000	0.8232	22(31.0%)	6(8.5%)	34(47.9%)	7(9.9%)	2(2.8%)
2024	0.4029	0.4574	0.2457	0.6098	0.0000	0.7912	17(23.9%)	4(5.6%)	34(47.9%)	16(22.5%)	0(0.0%)
**Table 8−2**. Official-list coverage and fixed-cohort diagnostics.											
**Metric**					**2010**	**2015**		**2019**	**2024**	**Change, 2010–2024**	
Counties with H>0,n					35	45		49	54	+19	
Counties with H=0, n					36	26		22	17	−19	
Full-sample meanD					0.1922	0.2849		0.3493	0.4029	+0.2108	
Fixed-support regional meanD					0.1922	0.2288		0.2596	0.2719	+0.0797	
Mean H in baseline-covered cohort					0.2122	0.2816		0.3102	0.3469	+0.1347	
Mean Q in baseline-covered cohort					0.1243	0.2018		0.3070	0.3281	+0.2037	
Mean C in baseline-covered cohort					0.9377	0.9419		0.9513	0.9475	+0.0097	
Mean M in baseline-covered cohort					0.1683	0.2417		0.3086	0.3375	+0.1692	
Mean D in baseline-covered cohort					0.3898	0.4642		0.5265	0.5516	+0.1617	
Mean |B| in baseline-covered cohort					0.2706	0.2502		0.2599	0.2630	−0.0075	
**Table 8−3**. Decomposition of the change in the regional mean D, 2010–2024.											
**Component**						**Contribution to** $\Delta \bar{D}$				**Share of total change**	
Change within the 35 counties covered in 2010						0.0797				37.8%	
Component associated with 19 post-2010 entrant counties						0.1310				62.2%	
Total change						0.2108				100.0%	

The decomposition is an accounting identity. The entrant component includes counties entering the official inventory after 2010 and the subsystem levels subsequently recorded in those counties; it is not a causal estimate of the effect of official listing.

Among the baseline-covered counties, mean D rose from 0.3898 to 0.5516 and mean M approximately doubled. Mean Q increased by 163.9%, compared with 63.5% for H, whereas C remained near 0.94–0.95 and |B| changed little. Paired tests confirmed significant increases in D and M both (p<0.001), with no significant changes in C (p=0.522) or |B| (p=0.645). Higher coordination within this cohort therefore came mainly from rising subsystem levels, particularly tourism development, while the relative mismatch between the two systems persisted.

Absolute disparities initially widened: the standard deviation increased from 0.2002 to 0.2532 by 2019 before declining to 0.2457 in 2024. The coefficient of variation fell from 1.0416 to 0.6098, partly because the regional mean increased and fewer counties remained at D=0. Severe and moderate imbalance declined from 81.7% to 29.5%, while transitional and moderate coordination rose from 18.3% to 70.4%. The principal change was broad middle-level upgrading; the disappearance of the two highly coordinated cases recorded in 2019 shows that a stable upper tier had yet to emerge.

#### 3.3.2 Spatial differentiation and lagging-system diagnosis.

Coupling coordination retained a marked core–periphery structure throughout the study period (Fig 8). Higher values were concentrated in southeastern Chongqing and western Hubei, with smaller nodes around Jishou–Huayuan, Songtao–Yanhe, and Cili. Low-value areas were more extensive in western Hunan and southern Guizhou. In 2024, mean D reached 0.699 among counties in the Chongqing section of the study area and 0.558 in the Hubei section, compared with 0.371 in Guizhou and 0.314 in Hunan. The difference of 0.385 between the Chongqing and Hunan sections indicates substantial geographical variation in the joint levels of the two standardized subsystems.

**Fig 8:**
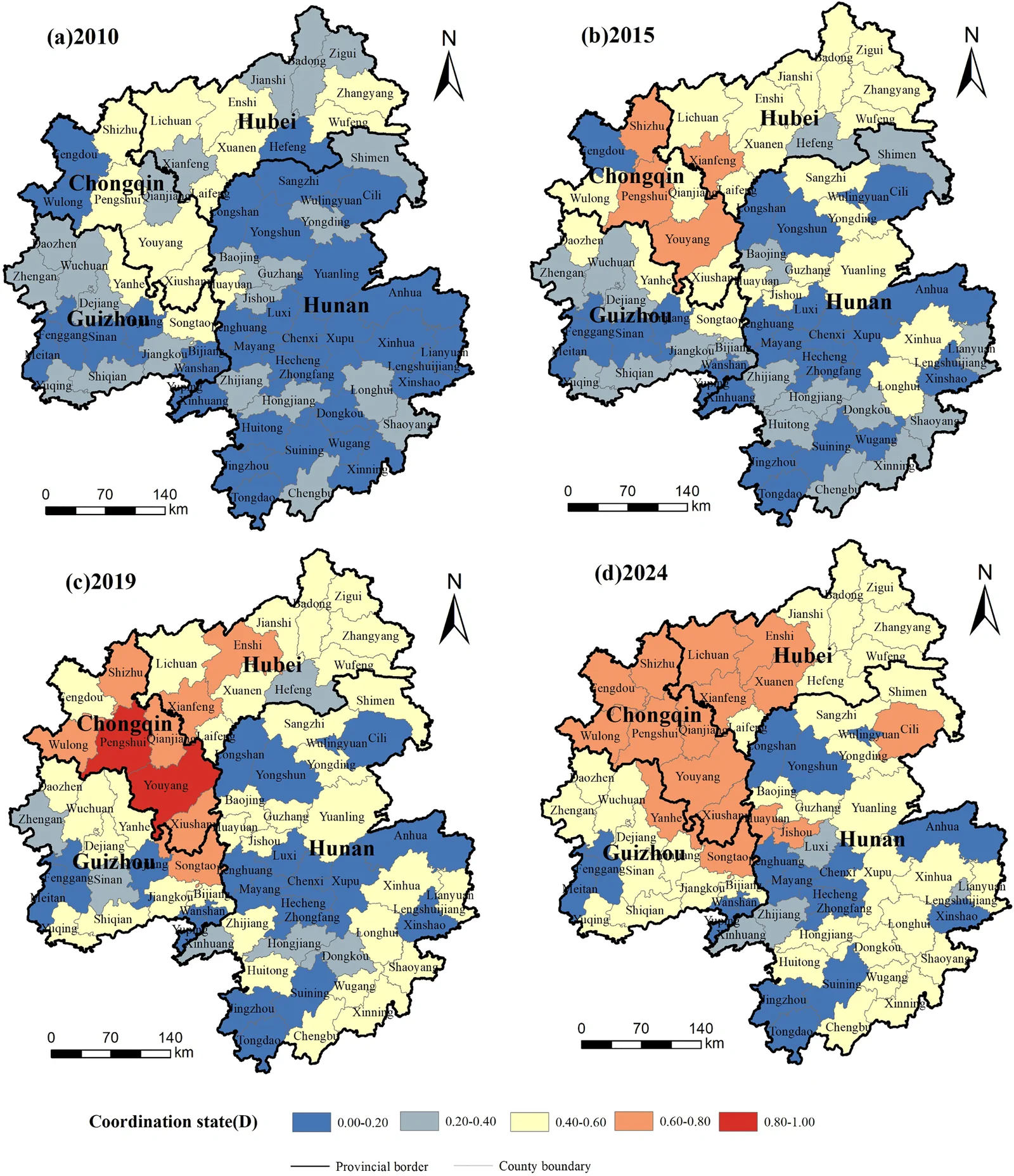
Spatial evolution of coupling coordination and lagging-system diagnosis in the Wuling Mountain Area, 2010–2024. Note: Administrative boundary data were obtained from the Standard Map Service System of the China Ministry of Natural Resources (http://bzdt.ch.mnr.gov.cn/), under the terms stated on the service website. The maps were created and edited by the authors. The administrative boundary data correspond to map review number GS(2024)0650. No Google Maps, Google Earth, Street View, satellite imagery, or other proprietary basemap was used.

The geography of D brings together differences in recorded SRICH endowment and tourism development. Under the equal-weight specification, D increases with the product of H and Q, so its spatial pattern reflects their joint configuration. Reading Fig 8 alongside the subsystem maps distinguishes counties with higher combined scores from those where one subsystem remains relatively lower. This comparison also clarifies why the geographical distribution of D differs from the distribution of either H or Q considered separately.

The relative-balance index shows that SRICH endowment was the more common lagging subsystem (Table 9). The share of counties with B<0 increased from 59.2% in 2010 to 74.6% in 2019, before declining to 67.6% in 2024. This change was driven by more than the reduction in zero-endowment counties. Counties with H = 0 fell from 36 to 17, whereas SRICH-lagging counties with a positive endowment increased from 6 in 2010–31 in 2019 and remained at 31 in 2024. The principal imbalance consequently shifted from an official-list coverage gap toward a difference in the growth rates of the two subsystems. Rapid tourism expansion during 2010–2019 raised Q faster than the batch-based accumulation of recognized SRICH, pushing the mean B down to −0.3468 in 2019. Between 2019 and 2024, slower tourism growth and renewed heritage recognition moved the mean to −0.2604 and increased the number of tourism-lagging counties from 18 to 23.

**Table 9:** Temporal distribution of the relative-balance index and subsystem-lag types, 2010–2024.

Year	Mean *B*	Median *B*	SRICH-lagging, (B < 0), n (%)	Tourism-lagging, (B > 0), n (%)	(H = 0), n (%)
2010	−0.3876	−1.0000	42 (59.2%)	29 (40.8%)	36 (50.7%)
2015	−0.3048	−0.1498	43 (60.6%)	28 (39.4%)	26 (36.6%)
2019	−0.3468	−0.2075	53 (74.6%)	18 (25.4%)	22 (31.0%)
2024	−0.2604	−0.1819	48 (67.6%)	23 (32.4%)	17 (23.9%)

B<0 indicates a relative SRICH-endowment lag, whereas B>0 indicates a relative tourism-development lag. Counties with H=0 are reported separately.

The lagging types distinguish the relative positions of the standardized subsystem scores. Wulingyuan, Fenghuang, Yongding, and Enshi had higher Q than H, whereas Huayuan, Songtao, Yanhe, and Xianfeng showed the reverse configuration. The direction of B identifies the relatively lower subsystem, while describes their joint level. Used together, these measures distinguish the direction of imbalance from the overall level of the two scores, providing a more informative county diagnosis than the coordination class alone.

#### 3.3.3 Sensitivity analysis of coupling coordination.

The main results were highly stable under the two alternative weighting scenarios (Table 10). Regional mean D ranged from 0.1874 to 0.1967 in 2010 and from 0.4022 to 0.4033 in 2024, preserving the temporal pattern obtained with equal weights. Rank correlations with the baseline remained between 0.9940 and 0.9989, and 93.0–98.6% of counties retained their original coordination class. Mean absolute deviations were limited to 0.0051–0.0103, while the maximum deviation of 0.0465 was less than one quarter of the 0.20 class interval. Fig 9 likewise shows localized changes without altering the regional core–periphery structure. The temporal pattern and county hierarchy remained stable across the tested subsystem-weight scenarios. Because H=0 fixes D=0 under every weighting scenario, the coverage effect was examined separately in Section 3.3.1.

**Table 10:** Sensitivity of coupling coordination to alternative subsystem weights.

Table 10−1. Continuous values and county rankings.									
Year	Mean *D*: Baseline	Mean D: SRICH-priority	Mean D: Tourism-priority	Mean |ΔD|: SRICH	Mean |ΔD|: Tourism	Max |ΔD|: SRICH	Max |ΔD|: Tourism	Spearman ρ: SRICH	Spearman ρ: Tourism
2010	0.1922	0.1967	0.1874	0.0051	0.0053	0.0288	0.0309	0.9989	0.9980
2015	0.2849	0.2877	0.2819	0.0070	0.0071	0.0327	0.0350	0.9967	0.9975
2019	0.3493	0.3475	0.3508	0.0089	0.0089	0.0320	0.0341	0.9988	0.9963
2024	0.4029	0.4022	0.4033	0.0103	0.0103	0.0435	0.0465	0.9968	0.9940
**Table 10−2** **. Stability of coordination classes.**									
**Year**	**Same class: SRICH, n (%)**	**Same class: tourism, n (%)**	**Upgrade: SRICH n**	**Downgrade: SRICH n**		**Upgrade: Tourism n**		**Downgrade: Tourism n**	**Structural-zero n**
2010	70 (98.6%)	68 (95.8%)	1	0		0		3	36
2015	69 (97.2%)	69 (97.2%)	1	1		0		2	26
2019	66 (93.0%)	69 (97.2%)	2	3		2		0	22
2024	70 (98.6%)	67 (94.4%)	1	0		3		1	17

The baseline specification uses equal subsystem weights. The SRICH-priority and tourism-priority specifications assign weights of 0.6 and 0.4 in opposite directions.

Structural-zero counties are those with H=0, for which D=0 under every weighting scenario.

**Fig 9:**
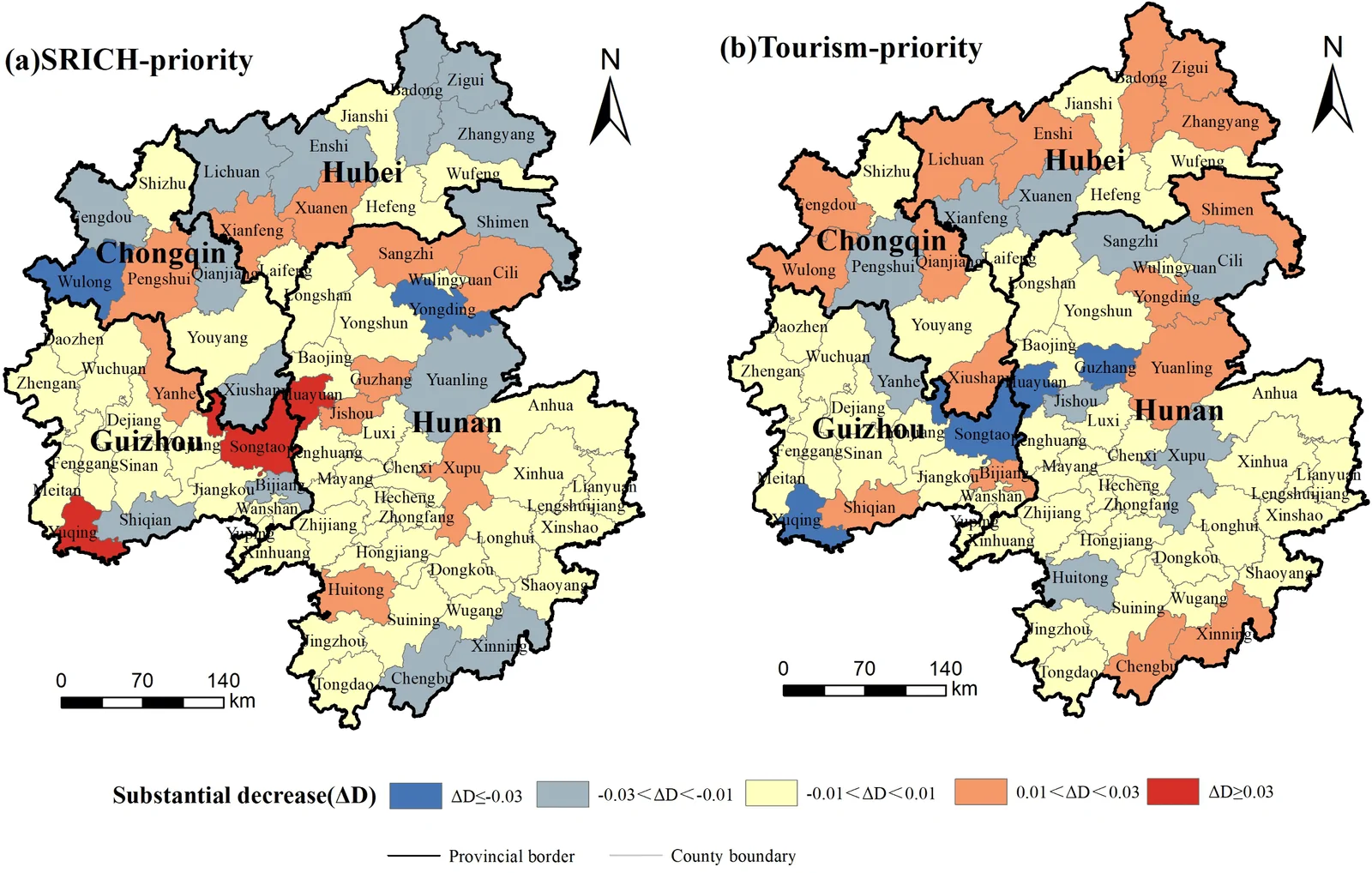
Sensitivity of county-level coupling coordination to alternative subsystem weights, 2010–2024. Note: Administrative boundary data were obtained from the Standard Map Service System of the China Ministry of Natural Resources (http://bzdt.ch.mnr.gov.cn/), under the terms stated on the service website. The maps were created and edited by the authors. The administrative boundary data correspond to map review number GS(2024)0650. No Google Maps, Google Earth, Street View, satellite imagery, or other proprietary basemap was used.

The direction of the small deviations provides further insight into subsystem change. Alternative weights affect M, while C remains constant. Relative to the baseline, SRICH priority changes the combined development index by 0.1(H−Q), and tourism priority changes it by 0.1(Q−H). Accordingly, the SRICH-priority scenario produced the higher regional mean in 2010 and 2015, whereas tourism priority produced the higher mean in 2019 and 2024. The reversal in the regional response to weighting is consistent with the changing relative positions of H and Q reported in Table 9. It shows that the small differences between weighting scenarios contain information about subsystem asymmetry, even while the overall rankings remain stable.

Class changes occurred mainly where pronounced subsystem imbalance coincided with proximity to a fixed threshold. In 2024, SRICH priority raised Pengshui from 0.7912 to 0.8015, moving it into high coordination. Tourism priority moved Changyang, Zhijiang, and Luxi into higher classes, while Yanhe fell below the moderate-coordination threshold. These shifts followed the leading subsystem: Pengshui and Yanhe were more responsive to the weight assigned to SRICH, whereas the other three counties benefited from greater tourism weight. Only one to five counties changed class in any scenario–year combination, confirming that threshold sensitivity was confined to a small set of marginal cases. For counties close to a class boundary, continuous D values and the direction of B provide a clearer interpretation of their subsystem configuration than the class label alone.

### 3.4 Global and local Moran’s I

#### 3.4.1 Global spatial dependence.

SRICH endowment, tourism development, and coupling coordination exhibited significant positive spatial autocorrelation in all four benchmark years (Table 11 and Fig 10). Global Moran’s I ranged from 0.1823 to 0.5107, with permutation-based p-values no greater than 0.019. Under the queen-contiguity specification, neighboring counties tended to have similar index values.

**Table 11:** Global Moran’s I for SRICH endowment, tourism development, and coupling coordination, 2010–2024.

Year	H: Moran’s (I)	H: pseudo-(p)	Q: Moran’s (I)	Q: pseudo-(p)	D: Moran’s (I)	D: pseudo-(p)
2010	0.1823	0.019	0.4506	0.001	0.2871	0.002
2015	0.2867	0.002	0.5107	0.001	0.296	0.001
2019	0.321	0.001	0.31	0.002	0.3398	0.001
2024	0.1958	0.011	0.4374	0.001	0.3217	0.001

Spatial relationships are based on a row-standardized first-order queen-contiguity matrix. Pseudo-p values are based on 999 random permutations.

**Fig 10:**
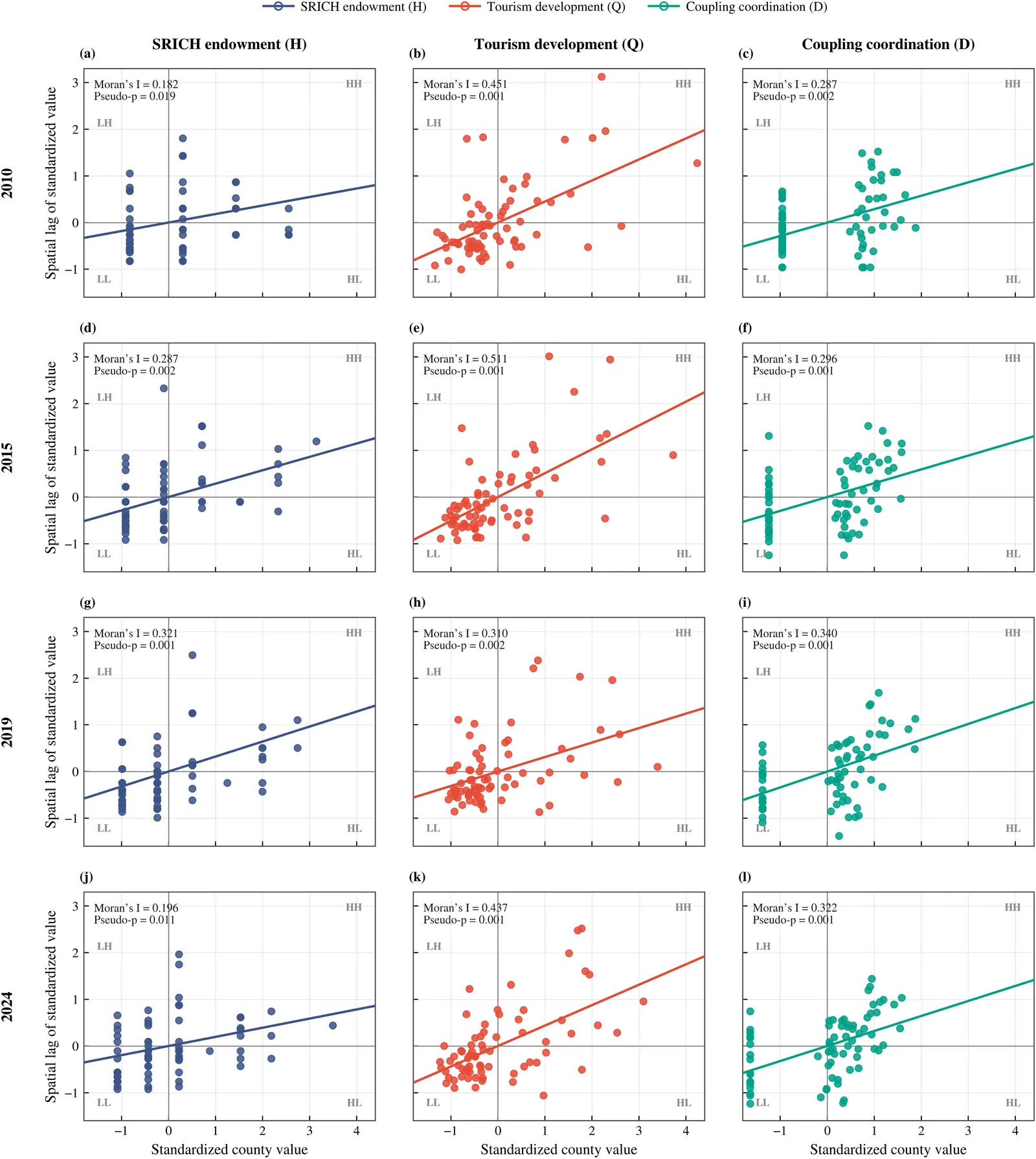
Moran scatterplots for SRICH endowment, tourism development, and coupling coordination in the Wuling Mountain Area, 2010–2024. Note: H denotes SRICH endowment, Q denotes tourism development, and D denotes coupling coordination. Spatial relationships are based on the row-standardized first-order queen-contiguity matrix.

The strength of this association varied across subsystems and years. Moran’s I for H increased from 0.1823 in 2010 to 0.3210 in 2019, before declining to 0.1958 in 2024. The later decline accompanied broader official-list coverage and lower geographical concentration. Q showed stronger spatial autocorrelation than H in every benchmark year: its Moran’s I increased from 0.4506 in 2010 to 0.5107 in 2015, fell to 0.3100 in 2019, and recovered to 0.4374 in 2024. The two subsystems therefore differed both in the strength of neighboring similarity and in its temporal variation.

Moran’s I for D increased from 0.2871 in 2010 to 0.3398 in 2019 and then declined to 0.3217 in 2024. Its value exceeded those of both subsystems in 2019, indicating that the composite score had a distinct pattern of spatial association in that year. Together, these statistics describe regional differences in neighboring similarity; their local distribution is examined in Section 3.4.2.

#### 3.4.2 Local clusters and cross-system spatial correspondence.

FDR-adjusted local results identified a small number of significant counties (Fig 11 and Table 12). In 2010, three counties were significant for H and five for D; three significant D observations remained in 2015. In 2019, three counties were significant for Q and two for D. None passed the adjusted threshold in 2024. Global Moran’s I summarizes association across the regional contiguity structure, while the adjusted local tests identify individual counties with significant local configurations. The results therefore combine persistent regional association with sparse and temporally variable local significance.

**Table 12:** FDR-adjusted local Moran cluster counts, 2010–2024.

Year	System	HH	LL	HL	LH	FDR q < 0.05	Raw p < 0.05
2010	H	0	0	3	0	3	12
2010	Q	0	0	0	0	0	14
2010	D	2	0	3	0	5	10
2015	H	0	0	0	0	0	11
2015	Q	0	0	0	0	0	16
2015	D	2	0	1	0	3	12
2019	H	0	0	0	0	0	17
2019	Q	2	1	0	0	3	12
2019	D	1	0	1	0	2	17
2024	H	0	0	0	0	0	12
2024	Q	0	0	0	0	0	14
2024	D	0	0	0	0	0	18

HH and LL denote high–high and low–low clusters, respectively; HL and LH denote high–low and low–high spatial outliers. FDR-adjusted counts are based on the Benjamini–Hochberg correction across the 71 local tests for each system–year combination.

**Fig 11:**
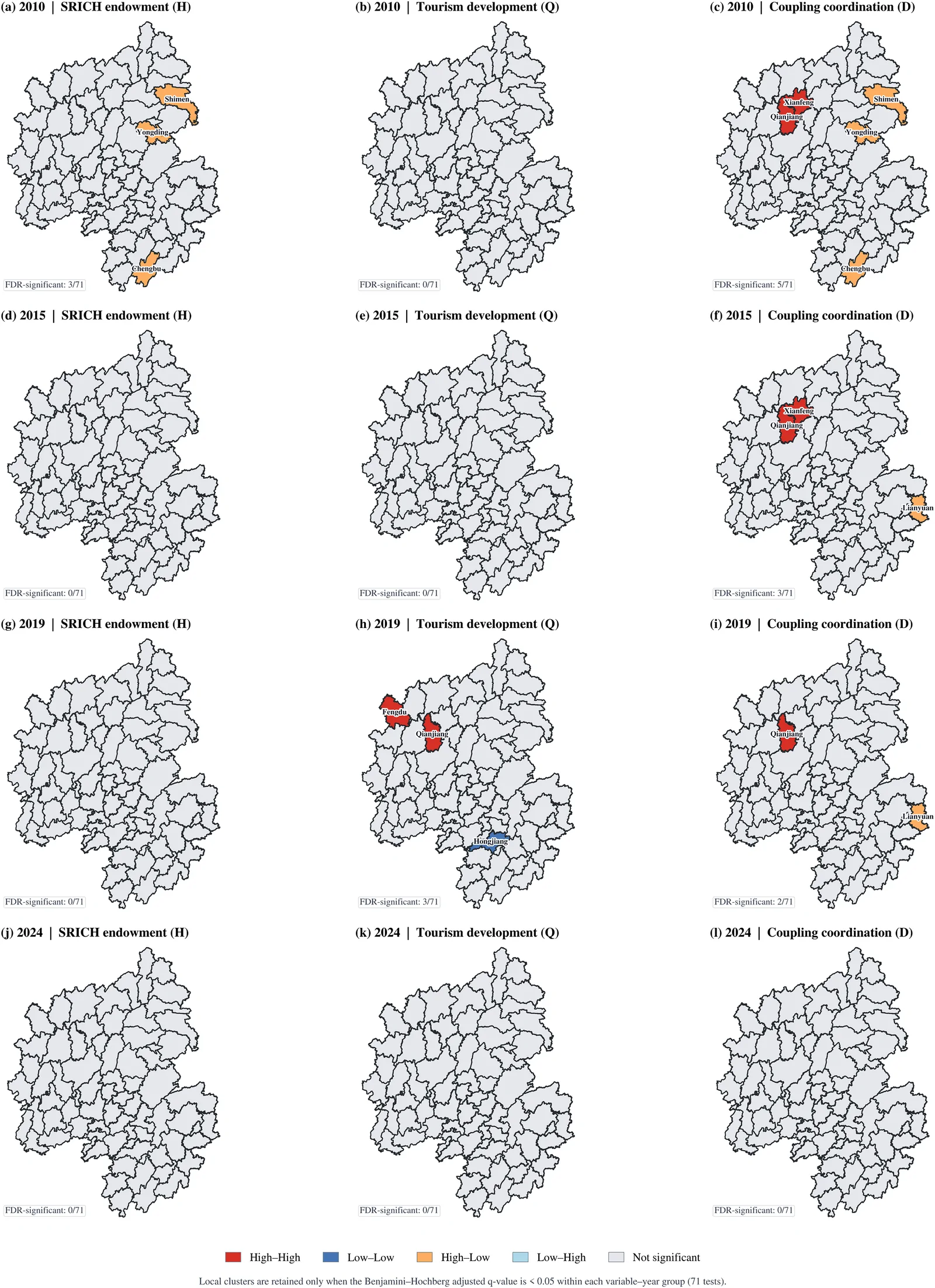
Temporal evolution of FDR-adjusted local Moran cluster types for SRICH endowment, tourism development, and coupling coordination in the Wuling Mountain Area, 2010–2024. Note: Colored counties satisfy the FDR-adjusted significance criterion q<0.05 after system- and year-specific Benjamini–Hochberg correction. Gray counties are not significant after correction. Administrative boundary data were obtained from the Standard Map Service System of the China Ministry of Natural Resources (http://bzdt.ch.mnr.gov.cn/), under the terms stated on the service website. The maps were created and edited by the authors. The administrative boundary data correspond to map review number GS(2024)0650. No Google Maps, Google Earth, Street View, satellite imagery, or other proprietary basemap was used.

In 2010, Yongding, Shimen, and Chengbu were HL outliers for both H and D, giving an H−D Jaccard coefficient of 0.60 (Fig 12 and Table 13). These counties combined relatively high own values with lower neighboring values in both indices. Qianjiang and Xianfeng were HH observations for D in both 2010 and 2015, identifying a recurring local configuration across the Chongqing–Hubei boundary. Lianyuan was an HL outlier in 2015. These cases distinguish neighboring concentrations of higher scores from individual high-value counties surrounded by lower values.

**Table 13:** Non-zero correspondence between FDR-significant local-cluster sets.

Year	Pair	Sig. A	Sig. B	Both	Same type	Union	Jaccard
2010	H−D	3	5	3	3	5	0.60
2019	Q−D	3	2	1	1	4	0.25

The Jaccard coefficient is calculated as the intersection divided by the union of the two FDR-significant county sets.

**Fig 12:**
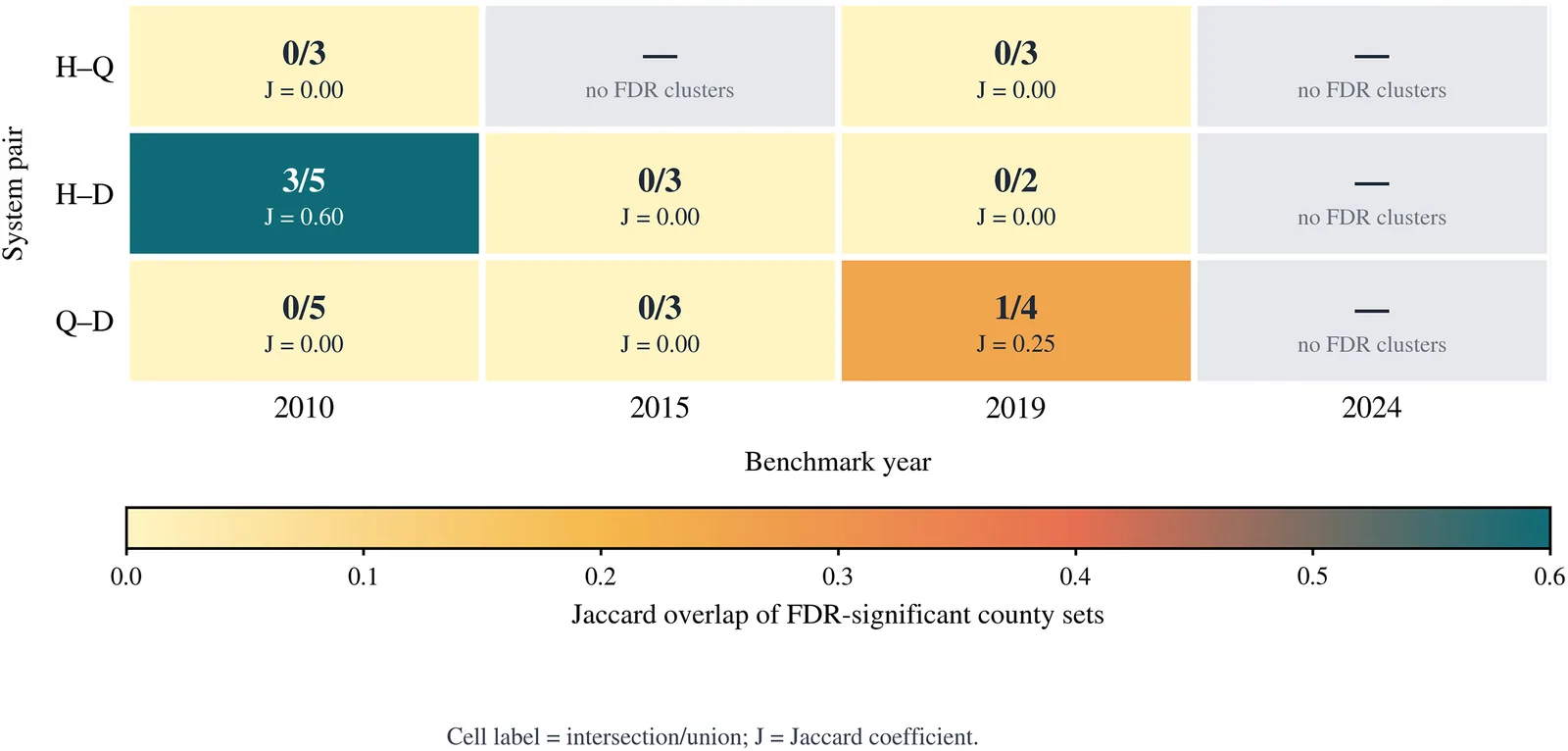
Cross-system correspondence of FDR-significant local-cluster sets, 2010–2024. Note: Each cell reports the intersection and union of the corresponding FDR-significant county sets, with color indicating the Jaccard coefficient. A dash indicates that neither member of the system pair contained an FDR-significant county in that year.

In 2019, Fengdu and Qianjiang were HH observations for Q, while Hongjiang was LL. For D, Qianjiang remained HH and Lianyuan remained HL; no H observation passed FDR correction. Qianjiang was the only county with the same significant type in Q and D, producing a Q−D Jaccard coefficient of 0.25. Hongjiang’s LL result for Q had no corresponding significant D classification. The overlap was therefore confined to selected counties, with the composite index displaying a local pattern distinct from either subsystem.

Although 12–18 counties per system met the unadjusted p<0.05 threshold in 2024, none remained significant after FDR correction. Cross-system correspondence also varied across benchmark years, from the H−D overlap in 2010 to the Q−D overlap in 2019. Because D incorporates both subsystem scores, these overlaps describe the geographical correspondence of related measures. Overall, the adjusted results identify a limited set of recurring clusters and outliers within a region that retained significant global spatial association.

## 4. Discussion

### 4.1 Expanding recognition within a stable administrative geography

The SRICH inventory expanded across the Wuling Mountain Area while retaining a recognizable spatial structure. From 2010 to 2024, the number of projects increased from 45 to 110, county coverage rose from 35 to 54, and the geographical concentration index declined from 18.64 to 16.12. The decline in concentration is consistent with recognition reaching more counties, whereas the stronger KDE peaks indicate that many newly recognized projects remained near established project locations. The broadly stable SDE envelope suggests that the overall regional range changed little during this expansion. Performance-based heritage remained the largest category, while competitive and ritual–festival practices grew more rapidly. Their overlap reflects the composite character of local traditions, in which bodily skills, performance, ritual, and collective memory can coexist. These indicators describe the changing distribution of officially recognized projects, while the practice of SRICH may extend beyond the recorded locations.

The persistence of established concentrations is consistent with the place-based conditions under which SRICH is practiced and transmitted. Embodied learning, repeated participation, ritual calendars, and access to communal spaces can help maintain local continuity, while markets, migration, intermarriage, and festivals provide occasions for interaction among neighboring communities [15,39]. In the Wuling ethnic corridor, the shared cultural landscape of Tujia, Miao, Dong, and other groups provides a relevant context for interpreting the coexistence of stable spatial boundaries, denser cores, and wider county coverage. The spatial pattern thus reflects the joint presence of local continuity and regional cultural interaction, while the indicators themselves primarily reveal the geography of formal recognition.

Official inventories also constitute a particular form of heritage representation [19]. In this study, recent additions were concentrated in Hunan and provincial-level lists, while the absence of additions in several years showed the uneven timing of inventory growth. County comparisons should therefore consider both cumulative recognized stocks and the timing of additions. Cross-provincial surveys and community verification could complement these records with information on active practices, while monitoring transmission frequency, bearer continuity, participation, and practice spaces would connect inventory review with safeguarding priorities.

### 4.2 Tourism growth within a persistent county hierarchy

Tourism development increased substantially, while a persistent county hierarchy remained evident. Mean Q rose from 0.1118 in 2010 to 0.2892 in 2024, with most growth occurring before 2019. The subsequent decline in the coefficient of variation from 0.5636 to 0.5034 coincided with a faster increase in the median than in the mean and a lower maximum, indicating improvement among middle-ranking counties and some compression at the upper end. Nevertheless, only 24 counties exceeded the regional mean in 2024, and established centers in southeastern Chongqing, southwestern Hubei, and northwestern Hunan retained prominent positions. The narrowing of disparities therefore accompanied a broader distribution of tourism development within a largely persistent spatial hierarchy.

This persistence is consistent with the destination trajectories discussed in research on path dependence [60]. Previous studies have examined spatially uneven relationships between transport improvement and tourism growth [61], the association of accessibility with tourism attractiveness in mountainous settings [62], and differences in accessibility and tourism development following infrastructure expansion [63,64]. These findings provide context for the county hierarchy observed here. Its interpretation also depends on the composition of Q: tourist arrivals and tourism revenue account for 62.16% of the entropy weights, while road mileage contributes 11.34%. The index consequently emphasizes realized market scale alongside tourism supply and supporting capacity. Positive Moran’s I further indicates similarity among neighboring counties, complementing the evidence of regional centers and lower-value areas. Together, these results describe an uneven geography of measured tourism development.

The coexistence of established centers and numerous counties below the regional mean suggests a basis for differentiated regional planning. Cross-provincial coordination could prioritize compatible visitor information, service standards, and itinerary planning, while investment decisions should reflect local gaps in access and tourism services. Research on cross-border tourism governance highlights the importance of aligning responsibilities across territorial levels [65]. For the Wuling Mountain Area, clearly assigned responsibilities and shared monitoring could provide a practical framework for joint planning. Alongside arrivals and revenue, evaluation could include overnight stays, multi-county visits, local procurement, and community income to assess how tourism activity is distributed. These priorities follow from the observed county hierarchy, with specific interventions requiring assessment of local needs.

### 4.3 Rising coordination, persistent imbalance

The regional mean D rose from 0.192 in 2010 to 0.403 in 2024. Of the total increase of 0.2108, counties entering the official SRICH inventory after 2010 accounted for 62.2%, while the 35 counties already covered in 2010 accounted for 37.8%. The number of counties with H=0 fell from 36 to 17. Within the fixed 2010 cohort, mean D increased from 0.390 to 0.552 and mean M approximately doubled, whereas mean C remained close to 0.94–0.95 and mean |B| changed only slightly. The increases in D and M were statistically significant (p<0.001), while the changes in C (p=0.522) and |B| p=0.645 were not. Under the equal-weight specification, D increases with the joint product of H and Q. Thus, a higher D value can result from higher combined subsystem levels even when their relative balance changes little. County rankings and coordination classes remained highly stable under the tested alternative weights. The increase in regional D therefore reflects wider official-list coverage and higher joint subsystem levels more clearly than an improvement in relative balance.

The two subsystems also changed at different rates. Between 2015 and 2019, mean Q increased by 0.099, compared with 0.028 for mean H, and the proportion of SRICH-lagging counties rose to 74.6%. After 2019, slower tourism growth and additional heritage recognition reduced the number of counties with the most pronounced recorded heritage deficit. The number of counties with positive but relatively lower H, however, remained substantial. This indicates that the imbalance increasingly concerned differences in the growth rates of the two subsystem indices rather than the complete absence of recognized SRICH. Yongding, Wulingyuan, Fenghuang, and Enshi had lower H than Q, whereas Huayuan, Songtao, Yanhe, and Xianfeng showed the reverse configuration. These two groups represent different relative positions of the recorded SRICH and tourism-development indices. Research on Miao tourism communities in western Hunan shows that external investment can create livelihood opportunities while also raising concerns about local control and dependence [66]. This evidence supports the importance of considering community authority when county-level coordination is translated into project-level tourism planning.

Policy responses should reflect these different configurations. In tourism-developed counties with relatively low recorded H, project-level surveys, community verification, and transmission support can be used to assess whether additional SRICH recognition or tourism presentation is appropriate. In counties with relatively high H but lower Q, planning can first examine gaps in visitor access, interpretation, booking, safety, and links to established gateways. A regional mechanism could connect heritage surveys, nomination cycles, safeguarding plans, and tourism investment decisions across provincial boundaries. D can be used together with C and B to identify the direction and degree of county-level imbalance, while project-level assessment can add evidence on transmission continuity, bearer participation, community authority, visitor learning, and locally retained benefits.

### 4.4 Differentiated pathways toward sustainable SRICH–tourism integration

Tourism planning for SRICH should prioritize compatibility with the social and cultural conditions through which practices are maintained and transmitted [3,21]. Performance-based, competitive, and ritual–festival heritage differ in bodily participation, spatial dependence, seasonal rhythm, safety requirements, and the authority exercised by bearer communities. These categories may also overlap: dragon-boat racing, for example, can function simultaneously as a competition, a collective performance, and an event embedded in a ritual calendar. Planning should therefore consider the relationship among the practice, its bearer community, and its cultural setting. Where several cultural functions coexist, the most sensitive requirement should guide the intensity and form of tourism use. Formal recognition and county-level coordination scores provide regional context, whereas decisions about individual practices require project-level assessment and community participation.

Tourism use may create pressure when a practice is separated from its festival calendar, ritual space, agricultural cycle, or intercommunity relationships. It may also simplify complex movements, rules, training processes, and embodied knowledge into abbreviated forms designed for visitor convenience. External actors may further gain control over scheduling, interpretation, visual representation, or revenue, leaving bearer communities with limited influence over decisions. These concerns are consistent with wider discussions of authenticity, commodification, and practitioner participation in intangible heritage tourism [19–21]. At the same time, tourism can provide employment, visibility, and opportunities for cultural learning when local actors retain meaningful control over presentation and benefit distribution [66]. The county-level coordination score describes the joint level of two recorded subsystems, while cultural integrity, transmission quality, and community authority require project-level assessment.

Building on these considerations, project-level assessment could examine three linked dimensions: cultural compatibility, tourism capacity, and community decision-making authority. Cultural compatibility concerns whether a practice can be presented without undermining ritual meaning, restricted knowledge, or established forms of transmission. Tourism capacity includes seasonal availability, visitor numbers, safety, accessibility, and the ecological tolerance of the practice setting. Community authority concerns the ability of bearer groups to influence participation, interpretation, scheduling, and benefit allocation. A weakness in any one dimension would call for a narrower form of tourism use, ranging from limited interpretation to community-led experience or broader market connection. For Baishou dance and Maogusi, assessment could examine collective participation, presentation settings, and local narrative authority. For dragon-boat racing, stilt racing, and board-shoe racing, it could examine traditional rules, training, participant competence, and safety arrangements [67]. For firecracker snatching, fire-dragon customs, and dragon-lantern traditions, attention should be given to customary calendars, ritual spaces, and community decisions concerning restricted elements [68].Decisions about cultural access and representation should involve relevant knowledge holders and community groups.

Implementation can be supported through co-governance among bearer representatives, village collectives, cultural authorities, tourism operators, and safety specialists. Project agreements may specify control over scheduling, interpretation, visitor limits, benefit distribution, and temporary suspension when agreed safeguarding conditions are not met. A defined share of tourism income could support apprenticeships, youth training, equipment maintenance, and the conservation of practice spaces. Monitoring should combine visitor and economic indicators with evidence on transmission continuity, community authority, and locally retained benefits. These arrangements place continued practice and community decision-making at the center of tourism planning, while allowing the appropriate form and intensity of use to vary across practices and local settings.

## 5. Conclusions

This study examined the county-level geography of officially recognized sports-related intangible cultural heritage (SRICH) and its alignment with tourism development across 71 units in the Wuling Mountain Area in 2010, 2015, 2019, and 2024. Official SRICH coverage expanded from 35 to 54 counties, while the geographical concentration index declined from 18.64 to 16.12. Embodied Performance Heritage (S2) remained the largest analytical category. Under fixed KDE settings, absolute density peaks increased within a broadly stable regional ellipse; because the annual samples are cumulative and represented at county centers, this pattern reflects the administrative accumulation of official records rather than the diffusion or intensification of living practices. Tourism development increased most rapidly before 2019 and retained a marked county hierarchy.

Mean D rose from 0.1922 to 0.4029. The coverage decomposition attributes 62.2% of this increase to counties entering the official SRICH inventory after 2010 and 37.8% to changes within the 35 baseline-covered counties. Within that fixed cohort, D and M increased, whereas C and |B| changed little. The regional rise in D therefore reflected wider official-list coverage and higher joint subsystem levels more closely than closer relative balance. SRICH-lagging counties remained predominant. H, Q, and D all showed significant positive global spatial autocorrelation, while FDR-stable local clusters were sparse and none survived correction in 2024.

These results support differentiated, cross-provincial governance. Heritage-rich counties with weaker tourism systems need basic access, interpretation, safety, and market links; mature destinations with limited formally recorded SRICH need community-based surveys and safeguarding assessment before tourism use is pursued. The findings remain bounded using official project stocks, aggregate tourism indicators, four benchmark years, county-level aggregation, and a single spatial-weight specification.

Because tourism revenue enters directly into X2 and also forms the numerator of X6, the composite tourism index gives weight to both absolute tourism revenue and its ratio to county GDP. This measurement feature should be considered when interpreting the index. Coupling coordination and Moran statistics describe comparative alignment and spatial association; they do not identify causal effects or establish sustainable integration. Future work should add indicators of transmission vitality, community authority, local benefit retention, visitor learning, and ecological pressure, together with annual data and designs capable of evaluating policy and infrastructure effects.

## Supporting information

S1 File Underlying research dataset. County-level tourism development indicators for 71 counties in the Wuling Mountain Area across four benchmark years (2010, 2015, 2019, and 2024), together with 118 county–project records of sports-related intangible cultural heritage.
(XLSX)

## References

[1] Qiu Q, Zuo Y, Zhang M. Intangible cultural heritage in tourism: Research review and investigation of future agenda. Land. 2022;11(1):139. doi: 10.3390/land11010139

[2] Unesco I. Basic texts of the 2003 convention for the safeguarding of the intangible cultural heritage. 2020 ed. Paris: UNESCO; 2020 [cited 2026 Aug 24]. Available from: https://unesdoc.unesco.org/ark:/48223/pf0000379091?3=null&queryId=e6e30211-a865-424e-a7c6-623bc1168ea6

[3] Huck PDW. Sustainable development goals: Article-by-article commentary. Nomos Verlagsgesellschaft mbH & Co. KG; 2022. doi: 10.5771/9783748902065

[4] Lee BX, Kjaerulf F, Turner S, Cohen L, Donnelly PD, Muggah R, et al. Transforming our world: Implementing the 2030 agenda through sustainable development goal indicators. J Public Health Policy. 2016;37 Suppl 1:13–31. doi: 10.1057/s41271-016-0002-7 27638240

[5] China’s emerging legislative and policy framework for safeguarding intangible cultural heritage. Int J Cult Policy. [cited 2026 Aug 26]. Available from: https://www.tandfonline.com/doi/abs/10.1080/10286632.2021.1993838

[6] Maags C. State institutions as building blocks of china’s infrastructures of memory – The case of intangible heritage. J Curr Chin Aff. 2023;52(2):163–84. doi: 10.1177/18681026221145950

[7] Mo B, Zhang X, Chen W, Zhou R, Zhang J. Research on the spatial distribution characteristics and influencing factors of sports intangible cultural heritage in the Guangdong-Hong Kong-Macao Greater Bay Area. Sci Rep. 2025;15(1):27147. doi: 10.1038/s41598-025-10734-3 40715224 PMC12296734

[8] Xiaorong C. Sports intangible cultural heritage: Concept, characteristic, function, classification. China Sport Sci. 2022;42:14–21. doi: 10.16469/j.css.202204002

[9] Ramshaw G, Gammon S. More than just nostalgia? Exploring the heritage/sport tourism Nexus. J Sport Tour. 2005;10(4):229–41. doi: 10.1080/14775080600805416

[10] Xie PF. The bamboo-beating dance in Hainan, China: Authenticity and commodification. J Sustain Tour. 2003;11(1):5–16. doi: 10.1080/09669580308667190

[11] Zhu Y. Performing heritage: Rethinking authenticity in tourism. Ann Tour Res. 2012;39(3):1495–513. doi: 10.1016/j.annals.2012.04.003

[12] Yang J, Ryan C, Zhang L. Impersonation in ethnic tourism – The presentation of culture by other ethnic groups. Ann Tour Res. 2016;56:16–31. doi: 10.1016/j.annals.2015.10.005

[13] Esfehani MH, Albrecht JN. Planning for intangible cultural heritage in tourism: Challenges and implications. J Hosp Tour Res. 2019;43(7):980–1001. doi: 10.1177/1096348019840789

[14] Alienation and authenticity in intangible cultural heritage tourism production. [cited 2026 Aug 26]. Available from: https://onlinelibrary.wiley.com/doi/epdf/10.1002/jtr.2478

[15] Li G, Zhang B. Identification of landscape character types for trans-regional integration in the Wuling Mountain multi-ethnic area of southwest China. Landsc Urban Plan. 2017;162:25–35. doi: 10.1016/j.landurbplan.2017.01.008

[16] Wu J, Wang K, He L, Chen B, Liu S. Coupling coordination degree between residents’ livelihoods and ecological resilience in the Wuling Mountain Area. iScience. 2026;29(6):115851. doi: 10.1016/j.isci.2026.115851 42181276 PMC13191300

[17] Zeng X, Yu L, Fang L, Tian G, Zhang H, Zhang Y. The spatiotemporal evolution and associated factors of Chinese traditional sports, games, and acrobatics intangible cultural heritage. Geocarto Int. 2026;41(1):2690364. doi: 10.1080/10106049.2026.2690364

[18] Tan N, Anwar S, Jiang W. Intangible cultural heritage listing and tourism growth in China. J Tour Cult Change. 2022;21(2):188–206. doi: 10.1080/14766825.2022.2068373

[19] Kirshenblatt-gimblett B. Intangible heritage as metacultural Production1. Mus Int. 2004;56(1–2):52–65. doi: 10.1111/j.1350-0775.2004.00458.x

[20] Cohen E. Authenticity and commoditization in tourism. Ann Tour Res. 1988;15(3):371–86. doi: 10.1016/0160-7383(88)90028-x

[21] Kim S, Whitford M, Arcodia C. Development of intangible cultural heritage as a sustainable tourism resource: The intangible cultural heritage practitioners’ perspectives. J Herit Tour. 2019;14(5–6):422–35. doi: 10.1080/1743873x.2018.1561703

[22] Kou W, Zhai J. Spatial distribution patterns and influencing factors of sports intangible cultural heritage in China. Front Earth Sci. 2025;13:1556652. doi: 10.3389/feart.2025.1556652

[23] Chang B, Ding X, Xi J, Zhang R, Lv X. Spatial-temporal distribution pattern and tourism utilization potential of intangible cultural heritage resources in the Yellow River Basin. Sustainability. 2023;15(3):2611. doi: 10.3390/su15032611

[24] Ye P, Cai W, Jiang Y, Wang Y, Wang J. Mapping the cultural landscape: Spatio-temporal patterns and influences on sports intangible cultural heritage in the Yangtze River Delta, China. Geocarto Int. 2026;41(1):2666477. doi: 10.1080/10106049.2026.2666477

[25] Liu X. Analysis of the interconnected growth and driving factors of culture, sports, and tourism industries in Guizhou Province, China. J Hosp Tour Insights. 2026;9:256–75. doi: 10.1108/JHTI-10-2025-1151

[26] Yuan C, Gan L, Zhuo H. Coupling mechanisms and development patterns of revitalizing intangible cultural heritage by integrating cultural tourism: The case of Hunan Province, China. Sustainability. 2022;14(12):6994. doi: 10.3390/su14126994

[27] Geng Y, Zhu H, Zhu R. Coupling coordination between cultural heritage protection and tourism development: The case of China. Sustainability. 2022;14(22):15421. doi: 10.3390/su142215421

[28] Du Y, Chen L, Xu J. Interactive effects of intangible cultural heritage and tourism development: A study based on the data panel PVAR model and coupled coordination model. Herit Sci. 2024;12:401. doi: 10.1186/s40494-024-01502-z

[29] Shao D, Zoh K, Xie Y. The spatial differentiation mechanism of intangible cultural heritage and its integration with tourism development based on explainable machine learning and coupled coordination models: A case study of the Jiang-Zhe-Hu in China. Herit Sci. 2024;12(1):414. doi: 10.1186/s40494-024-01528-3

[30] Zhang Z, Cui Z, Fan T, Ruan S, Wu J. Spatial distribution of intangible cultural heritage resources in China and its influencing factors. Sci Rep. 2024;14(1):4960. doi: 10.1038/s41598-024-55454-2 38418517 PMC10902377

[31] Zuo Y, Qiu Q, Hu T, Zhang J. How natural environments influence traditional sports and games: A mixed methods study from China. Int Rev Sociol Sport. 2022;58(2):328–48. doi: 10.1177/10126902221096233

[32] Saura SC, Zimmermann AC. Traditional sports and games: Intercultural dialog, sustainability, and empowerment. Front Psychol. 2021;11:590301. doi: 10.3389/fpsyg.2020.590301 33584427 PMC7873946

[33] Fotheringham AS, Wong DWS. The modifiable areal unit problem in multivariate statistical analysis. Environ Plan A. 1991;23:1025–44. doi: 10.1068/a231025

[34] Liu C, Kang L. How do geographical factors affect the distribution of intangible cultural heritage: A case study of Xinjiang, China. Sustainability. 2023;15(11):8943. doi: 10.3390/su15118943

[35] Shen L, Huang Y, Huang Z, Lou Y, Ye G, Wong S-W. Improved coupling analysis on the coordination between socio-economy and carbon emission. Ecol Indic. 2018;94:357–66. doi: 10.1016/j.ecolind.2018.06.068

[36] Liu Z. Sustainable tourism development: A critique. J Sustain Tour. 2003;11(6):459–75. doi: 10.1080/09669580308667216

[37] Ge B, Song Y, Wang J, Wang Y, Yuan X. Spatio-temporal pattern and entropy variation of agricultural heritage in China. Herit Sci. 2024;12:415. doi: 10.1186/s40494-024-01541-6

[38] Li H, Hui Y, Pan J. Evolution and influencing factors of social-ecological system vulnerability in the Wuling Mountains Area. IJERPH. 2022;19(18):11688. doi: 10.3390/ijerph191811688 36141959 PMC9517326

[39] Wang ZF, Li Q, Wu W. Genome map construction of the cultural heritage landscape and its characteristics in traditional villages of Wuling Mountain Area. Econ Geogr. 2021;41:225–31. doi: 10.15957/j.cnki.jjdl.2021.11.025

[40] China Intangible Cultural Heritage Network and Digital Museum. National Representative List of Intangible Cultural Heritage. China Intangible Cultural Heritage Network and Digital Museum [Internet]. [cited 2026 Aug 26]. Available from: https://www.ihchina.cn/project.html?utm_source

[41] Ge Y, Yuan Y, Hu S, Ren Z, Wu Y. Space–time variability analysis of poverty alleviation performance in China’s poverty-stricken areas. Spat Stat. 2017;21:460–74. doi: 10.1016/j.spasta.2017.02.010

[42] Tian Y, Wang Z, Zhao J, Jiang X, Guo R. A geographical analysis of the poverty causes in China’s contiguous destitute areas. Sustainability. 2018;10(6):1895. doi: 10.3390/su10061895

[43] Huiqin L, Yujie H. The spatio-temporal evolution and influencing factors of common prosperity level in the Wuling Mountain Area. J Resour Ecol. 2024;15(6). doi: 10.5814/j.issn.1674-764x.2024.06.015

[44] Zhang Y, Hanna P, Vanclay F. Social impacts from tourism development in protected areas: Comparing communities in different conservation zones of Wulingyuan World Heritage Site, China. Land Use Policy. 2025;158:107753. doi: 10.1016/j.landusepol.2025.107753

[45] State Council of the People’s Republic of China. Notice on Publishing the Second Batch of the National Intangible Cultural Heritage List and the First Expanded List (Guo Fa [2008] No. 19). [cited 2026 Aug 26]. Available from: https://www.mct.gov.cn/whzx/bnsj/fwzwhycs/201111/t20111128_765125.html

[46] Gwerevende S, Mthombeni ZM. Safeguarding intangible cultural heritage: Exploring the synergies in the transmission of Indigenous languages, dance and music practices in Southern Africa. Int J Herit Stud. 2023;29(5):398–412. doi: 10.1080/13527258.2023.2193902

[47] Zhang Z, Qin J, Luo L, Feng Y. Research on the evaluation of coordinated development of tourism–economy–ecological environment along the silk road economic belt. Sustainability. 2022;14(21):13838. doi: 10.3390/su142113838

[48] Chen W, Yang L, Wu J, Wu J, Wang G, Bian J, et al. Spatio-temporal characteristics and influencing factors of traditional villages in the yangtze river basin: A geodetector model. Herit Sci. 2023;11:111. doi: 10.1186/s40494-023-00948-x

[49] Diggle P. A kernel method for smoothing point process data. Appl Stat. 1985;34(2):138. doi: 10.2307/2347366

[50] Zhang X, Xiang H, Liu R. Spatial pattern and influencing factors of intangible cultural heritage of music in xiangxi, central China. Herit Sci. 2022;10:39. doi: 10.1186/s40494-022-00672-y

[51] Lefever DW. Measuring geographic concentration by means of the standard deviational ellipse. Am J Sociol. 1926;32(1):88–94. doi: 10.1086/214027

[52] Zou Z, Yun Y, Sun J. Entropy method for determination of weight of evaluating indicators in fuzzy synthetic evaluation for water quality assessment. J Environ Sci (China). 2006;18(5):1020–3. doi: 10.1016/s1001-0742(06)60032-6 17278765

[53] Hou C, Zhang Y, Zhou X. Coupling coordination and influencing factors of intangible cultural heritage and tourism development: A case study of Sichuan province, China. Sustainability. 2026;18(6):2788. doi: 10.3390/su18062788

[54] Li Y, Li Y, Zhou Y, Shi Y, Zhu X. Investigation of a coupling model of coordination between urbanization and the environment. J Environ Manag. 2012;98:127–33. doi: 10.1016/j.jenvman.2011.12.025 22265813

[55] Pu L, Chen X, Jiang L, Zhang H. Spatiotemporal characteristics of coupling and coordination of cultural tourism and objective well-being in western China. IJERPH. 2022;20(1):650. doi: 10.3390/ijerph20010650 36612972 PMC9819302

[56] Moran PAP. Notes on continuous stochastic phenomena. Biometrika. 1950;37(1–2):17–23. doi: 10.2307/2332142 15420245

[57] Anselin L. Local indicators of spatial association—LISA. Geogr Anal. 1995;27(2):93–115. doi: 10.1111/j.1538-4632.1995.tb00338.x

[58] Anselin L, Syabri I, Kho Y. *GeoDa*: An introduction to spatial data analysis. Geogr Anal. 2005;38(1):5–22. doi: 10.1111/j.0016-7363.2005.00671.x

[59] Benjamini Y, Hochberg Y. Controlling the false discovery rate: A practical and powerful approach to multiple testing. J R Stat Soc: B (Methodol). 1995;57:289–300. doi: 10.1111/j.2517-6161.1995.tb02031.x

[60] Ma M, Hassink R. Path dependence and tourism area development: The case of Guilin, China. Tour Geogr. 2014;16(4):iv–iv. doi: 10.1080/14616688.2014.958296

[61] Tian F, Yang Y, Jiang L. Spatial spillover of transport improvement on tourism growth. Tour Econ. 2020;28(5):1416–32. doi: 10.1177/1354816620982787

[62] Dong B, Zhang M, Wang H, Wu J. Study on the interaction mechanism among spatial patterns of traditional villages and tourism attractiveness and accessibility in Guizhou province. Sci Rep. 2025;15(1):10365. doi: 10.1038/s41598-025-94514-z 40133547 PMC11937332

[63] Deng F, Tian Q, Arif M. Assessing the shifts in spatiotemporal ecotourism accessibility driven by high-speed rail development in China. Habitat Int. 2025;164:103514. doi: 10.1016/j.habitatint.2025.103514

[64] Cheng M, Yan R, Dang J. The siphoning effect of transportation infrastructure on tourism development: Evidence from China. Res Transp Bus Manag. 2025;62:101465. doi: 10.1016/j.rtbm.2025.101465

[65] Stoffelen A, Ioannides D, Vanneste D. Obstacles to achieving cross-border tourism governance: A multi-scalar approach focusing on the German-Czech borderlands. Ann Tour Res. 2017;64:126–38. doi: 10.1016/j.annals.2017.03.003

[66] Tian B, Stoffelen A, Vanclay F. Understanding resilience in ethnic tourism communities: The experiences of Miao villages in Hunan Province, China. J Sustain Tour. 2023;32(7):1433–52. doi: 10.1080/09669582.2023.2239525

[67] Luchoro-Parrilla R, Lavega-Burgués P, Damian-Silva S, Prat Q, Sáez de Ocáriz U, Ormo-Ribes E, et al. Traditional games as cultural heritage: The case of canary islands (Spain) from an ethnomotor perspective. Front Psychol. 2021;12:586238. doi: 10.3389/fpsyg.2021.586238 33716856 PMC7947194

[68] Forsyth M. Lifting the lid on “the community”: Who has the right to control access to traditional knowledge and expressions of culture? Int J Cult Prop. 2012;19:1–31. doi: 10.1017/S0940739112000021

